# Mineral phosphorus drives glacier algal blooms on the Greenland Ice Sheet

**DOI:** 10.1038/s41467-020-20627-w

**Published:** 2021-01-25

**Authors:** Jenine McCutcheon, Stefanie Lutz, Christopher Williamson, Joseph M. Cook, Andrew J. Tedstone, Aubry Vanderstraeten, Sasha Wilson, Anthony Stockdale, Steeve Bonneville, Alexandre M. Anesio, Marian L. Yallop, James B. McQuaid, Martyn Tranter, Liane G. Benning

**Affiliations:** 1 School of Earth & Environment, University of Leeds, Woodhouse Lane, Leeds, LS2 9JT UK; 2 Department of Earth and Environmental Sciences, University of Waterloo, Waterloo, N2L 3G1 ON Canada; 3 GFZ German Research Centre for Geosciences, Telegrafenberg, 14473 Potsdam Germany; 4 Bristol Glaciology Centre, University of Bristol, Bristol, BS8 1QU UK; 5 School of Biosciences, University of Bristol, Bristol, BS8 1TQ UK; 6 Institute of Biological, Environmental and Rural Sciences, Aberystwyth University, Aberystwyth, SY23 3DA UK; 7 Department of Geosciences, Environment and Society, Université Libre de Bruxelles, 1050 Bruxelles, Belgium; 8 Department of Earth and Atmospheric Sciences, University of Alberta, Edmonton, AB T6G 2E3 Canada; 9 Department of Environmental Science, Aarhus University, Frederiksborgvej 399, 4000 Roskilde, Denmark; 10 Department of Earth Sciences, Free University of Berlin, 12249 Berlin, Germany

**Keywords:** Element cycles, Element cycles

## Abstract

Melting of the Greenland Ice Sheet is a leading cause of land-ice mass loss and cryosphere-attributed sea level rise. Blooms of pigmented glacier ice algae lower ice albedo and accelerate surface melting in the ice sheet’s southwest sector. Although glacier ice algae cause up to 13% of the surface melting in this region, the controls on bloom development remain poorly understood. Here we show a direct link between mineral phosphorus in surface ice and glacier ice algae biomass through the quantification of solid and fluid phase phosphorus reservoirs in surface habitats across the southwest ablation zone of the ice sheet. We demonstrate that nutrients from mineral dust likely drive glacier ice algal growth, and thereby identify mineral dust as a secondary control on ice sheet melting.

## Abstract (web summary)

Melting of the Greenland Ice Sheet—a threat for sea level rise—is accelerated by ice algal blooms. Here the authors find a link between mineral phosphorus and glacier algae, indicating that dust-derived nutrients aid bloom development, thereby impacting ice sheet melting.

## Introduction

The Greenland Ice Sheet (GrIS) comprises only 11.2% of land ice on Earth^1^, yet surface melting and ice-calving from the GrIS accounted for 37% of cryosphere attributed sea level rise between 2012 and 2016^2^. Mass loss is predominantly determined by the incoming shortwave radiation flux^3,4^ modulated by surface albedo^5^. There has been a ~40% increase in surface melting and runoff from the GrIS over the last quarter of a century^6^ as a north−south oriented band of low-albedo ice, known as the Dark Zone, has developed along the western margin of the ice sheet^7^. Albedo depends on the physical structure of surface ice^8^ and the presence of light absorbing particulates (LAP), which include pigmented glacier snow and ice algae, black carbon (BC), and mineral dust^7^. Glacier ice algae (hereafter glacier algae) produce photoprotective phenolic pigments^9–14^ that lower ice sheet albedo on the landscape-scale, thereby contributing to melting^15,16^. Glacier algae were calculated to be directly responsible for 9−13% of the surface melting in the Dark Zone in 2016^16^, and there are comparable indirect effects due to water retention and changes to ice crystal fabric as a result of algal growth^8^. While glacier algal blooms can cover up to 78% of the ice surface^16^, they exhibit a high degree of interannual variability in intensity and spatial extent^7^ that is yet to be understood. Thus, there is a pressing need to better quantify the parameters that control glacier algal growth and constrain the impact of these blooms on ice sheet albedo, melting, and contributions to sea-level rise. Here we demonstrate, through nutrient addition experiments and spatially resolved mineralogical and geochemical data, that phosphorus is a limiting nutrient for glacier algae in the Dark Zone. We identify phosphorus-bearing minerals (hydroxylapatite) as the likely phosphorous nutrient source fueling glacier algal blooms. We also disentangle the biogeochemical controls on ice sheet darkening by characterizing the source, composition, and nutrient delivery capacity of mineral dust. These results, in combination with nutrient addition experiments, demonstrate that phosphorus from mineral dust is a limiting nutrient for algal blooms in the Dark Zone.

## Results and discussion

### Phosphorus is a limiting nutrient for glacier algae

**Fig. 1:**
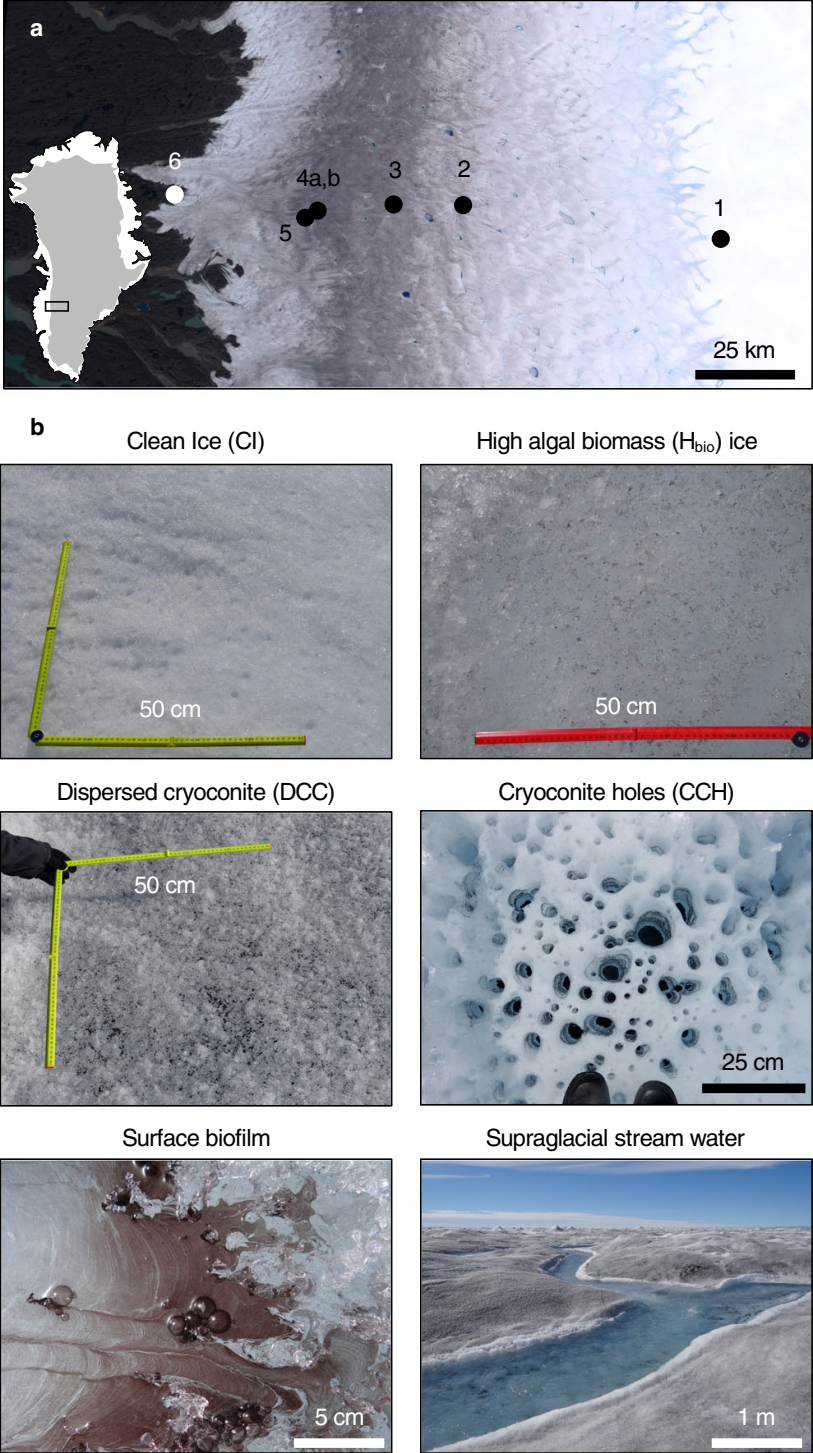
Sample collection locations and habitats on the Greenland Ice Sheet. **a** Sample collection sites 1–5 across the ablation zone in southwest Greenland Ice Sheet and rock sample collection site 6 near the terminus of Russell Glacier viewed using Sentinel-2 imagery (cloud-free composite of all acquisitions between 01/07/2016 and 31/08/2016); **b** photographs of surface ice habitats: clean ice (CI), high algal biomass ice (H_bio_), dispersed cryoconite ice (DCC), cryoconite holes (CCH), surface biofilm, and supraglacial stream water.

**Fig. 2:**
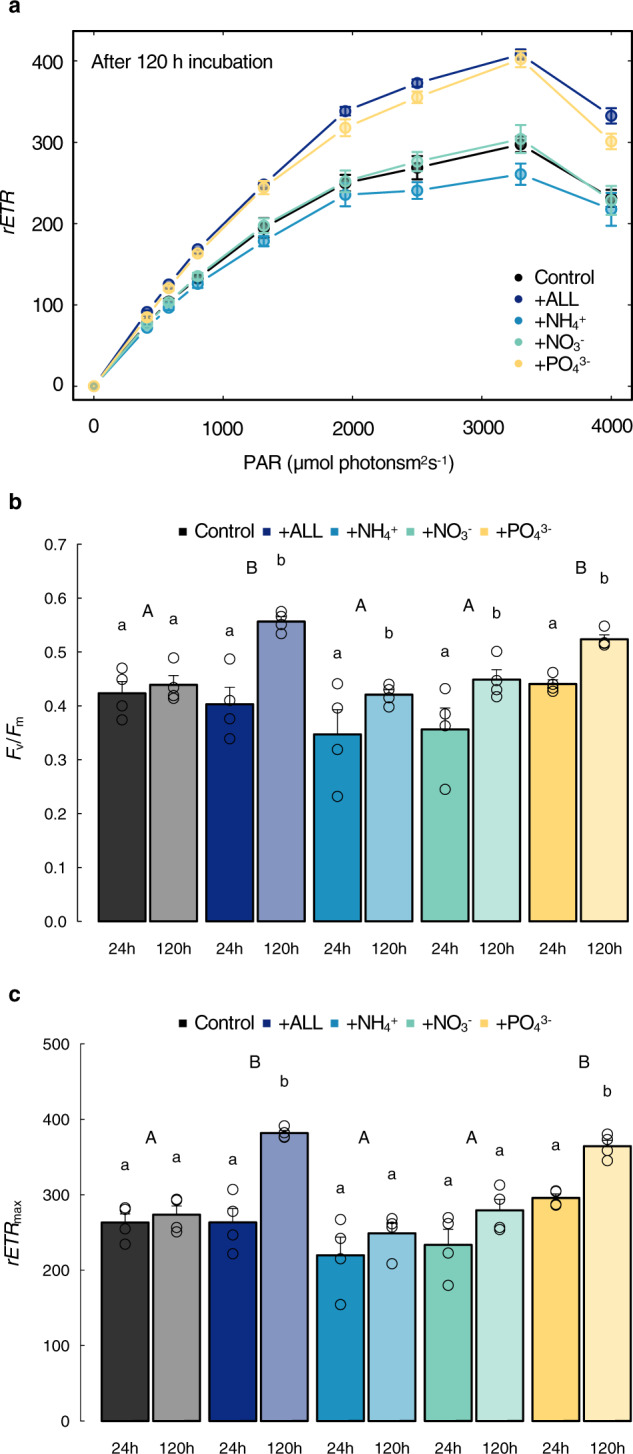
Glacier algal photophysiological response to nutrient addition. **a** Relative electron transport rates (*rETR*) measured during rapid light curves (RLCs) following 120 h incubation, **b** maximum quantum efficiency in the dark-adapted state (*F*_v_/*F*_m_) and **c** maximum electron transport rate (*rETR*_max_) after 24 and 120 h incubation. All plots show mean ±SE, *n* = 4. **b**, **c** lower and upper case letters denote homogeneous subsets identified from two-way ANOVA analysis of respective parameters in relation to duration and nutrient treatment, respectively. Two-way ANOVA results comparing treatments: *F*_4,30_ = 6.48, *P* = 0.000699, and time points (24 vs 120 h): *F*_4,30_ = 28.75, *P* = 0.00000839 for *F*_v_/*F*_m_. Two-way ANOVA results comparing treatments: *F*_4,30_ = 16.71, *P* = 0.000000264, and time points: *F*_4,30_ = 35.07, *P* = 0.00000174 for *rETR*_max_. The results of Tukey HSD tests comparing *F*_v_/*F*_m_ and *rETR*_max_ results for treatments at 24 and 120 h can be found in Supplementary Tables 2 and 3.

Glacier algal blooms were studied at five sites along a transect across the ablation zone in southwest GrIS (Fig. 1a). Surface snow and ice samples were collected at locations ~33−130 km from the ice margin in 2016 and 2017 (Sites 1−5), with reference rocks collected near the Russell Glacier terminus in 2018 (Site 6; Fig. 1a, Supplementary Table 1). Targeted surface habitats included clean ice (CI; free of macroscopically visible LAP), high algal biomass (H_bio_) ice, H_bio_ snow, dispersed cryoconite (DCC) ice, cryoconite holes (CCH), a floating biofilm, and supraglacial stream water (Fig. 1b). To identify potential nutrient limitations on glacier algal growth, we carried out a series of soluble nutrient addition incubation experiments at Site 4b using H_bio_ ice (8.0 ± 2.1 × 10^3^ cells mL^−1^) that was melted in the dark over 24 h, and re-incubated for 120 h across five treatments (phosphate, nitrate, ammonium, phosphate+nitrate+ammonium (+ALL), control). Concomitantly, the health and productivity of glacier algae assemblages were monitored using rapid light response curves^17^ performed with pulse amplitude modulation (PAM) fluorometery at 24, 72, 120 h. A significant response to phosphorus addition was apparent after 120 h of incubation (Fig. 2a), achieving the maximum quantum efficiency (*F*_v_/*F*_m_: inverse proxy of microalgae stress) and maximum rates of electron transport (*rETR*_max_: proxy for photosynthesis rate). Both parameters displayed positive responses to increased P availability (PO_4_^3-^ and +ALL treatments) compared to other treatments (Fig. 2b, c). Specifically, after 120 h there were no significant differences between the *rETR*_max_ measurements made for the phosphate and +ALL treatments, and both of these treatments were significantly higher than each of the control, ammonium, and nitrate treatments, with no significant differences between the latter three treatments (Supplementary Tables 2 and 3). These results indicate that phosphorus was the limiting nutrient for glacier algal growth at Site 4b. No significant differences in photophysiological parameters were apparent after 24 or 72 h across treatments (Supplementary Tables 2 and 3, Supplementary Fig. 1, data for 72 h not shown). The delayed response to phosphorus addition until 120 h suggests a mechanism of phosphate storage sufficient to sustain glacier algal productivity for ~5 days, which is similar in duration to the doubling time of 5.5 ± 1.7 days reported for glacier algal populations from this region^15^. Due to the slow doubling time of glacier algae, it is not surprising that a significant increase in cell counts was not measured over the 120 h experiment (Supplementary Table 4). Luxury uptake of phosphorus is common among microorganisms, with phosphorus stored intracellularly as polyphosphates that can act as an extraneous source under limiting conditions^18^. Such a storage mechanism would be beneficial for glacier algae inhabiting the oligotrophic surface ice environments of the GrIS Dark Zone. The lack of a photophysiological response to the addition of nitrate or ammonium suggests that glacier algae are not limited by N. Although our measurements of dissolved inorganic N in surface ice and snow samples were low or below detection limit (Supplementary Table 8), inorganic and organic N have been documented in H_bio_ ice habitats in concentrations of 1.0 and 14 µM, respectively^19^, indicating that N is available in the system.

### Phosphorus limitation decreases with increasing mineral phosphorus

**Fig. 3:**
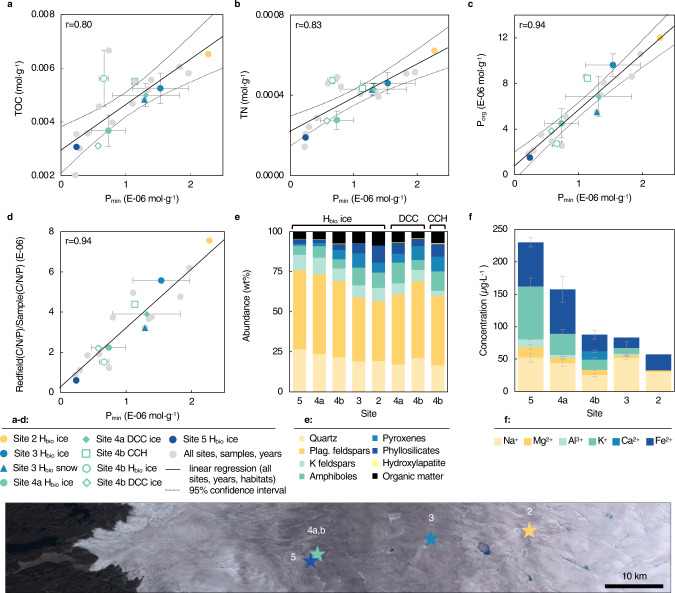
Dark Zone particulate matter nutrient concentrations, mineralogy, and major cation concentrations in associated meltwater. P_min_ concentration in particulates plotted against **a** total organic carbon (TOC), **b** total nitrogen (TN), **c** organic phosphorus (P_org_), and **d** molar C:N:P_org_ ratio normalized to the Redfield ratio (C:N:P 106:16:1). Linear regression and r-values (Pearson’s product-moment correlation) correspond to all data points from all sites (gray dots). **e** Relative mineral and organic matter abundances across the ablation zone, including hydroxylapatite (bright yellow); **f** major cation concentrations in meltwater from H_bio_ ice across the ablation zone. H_bio_ ice: high algal biomass ice; DCC ice: dispersed cryoconite ice; CCH: cryoconite hole. In **a**–**d**: colored points: mean values for different sites, habitats, and years, ±SE (2016: solid fill; 2017: white-fill); solid line: linear regression; thin dashed lines: 95% confidence interval. Site 2 H_bio_ ice *n* = 1; Site 3 H_bio_ ice *n* = 2; Site 3 H_bio_ snow *n* = 1; Site 4a H_bio_ ice *n* = 5; Site 4a DCC ice *n* = 2; Site 4b H_bio_ ice *n* = 2; Site 4b DCC ice *n* = 1; Site 4b CCH *n* = 1; Site 5 H_bio_ ice *n* = 1. In **e**: H_bio_ ice: site 5: *n* = 1; site 4a *n* = 5; site 4b: *n* = 4; site 3: *n* = 2; site 2 *n* = 1; DCC ice: site 4a *n* = 4; site 4b *n* = 1; CCH site 4b *n* = 1. In **f**: site 5 *n* = 6; site 4a *n* = 5; site 4b *n* = 3; site 3 *n* = 2; site 2 *n* = 1.

To assess potential phosphorus sources, sequential P extractions were conducted (Steps I, III, IV, and V from Ruttenberg, et al.^20^) and revealed that organic phosphorus (P_org_) accounted for up to 86% of the solid-phase P in H_bio_ ice (Supplementary Table 5), with exchangeable (P_exch_; <31%) and mineral phosphorus (P_min_; 17%) comprised the remaining solid-phase P. Molar concentrations of total organic carbon (TOC), total nitrogen (TN), and P_org_ all showed a positive correlation with particulate P_min_ concentrations (Fig. 3a–c, Supplementary Note 1, Supplementary Table 5). The solid-phase nutrient ratios (C:P_org_, C:N, and N:P_org_), which reflect the nutrient pools in the algal dominated biomass, indicate P as the limiting nutrient, particularly when compared to Redfield ratios (C:N:P 106:16:1; Supplementary Fig. 2)^21^. Normalizing solid-phase C:N:P molar ratios to Redfield C:N:P clearly shows that samples containing higher concentrations of P_min_ were closer to achieving Redfield ratio concentrations of organic nutrients, thereby providing an indication of potential nutrient limitation (Fig. 3d). The ratio of TOC:N:P_org_ measured in site 4 H_bio_ particulates ranged between 690:48:1 and 2615:196:1, which mirrors the dissolved organic nutrient ratios reported in Holland, et al.^19^ (DOC:DON:DOP = 2017:117:1). In the present study, we find that as the concentration of solid-phase P_min_ increases, the C:N:P ratio decreases and approaches the ideal Redfield C:N:P ratio. This trend was observed for solid-phase samples from all habitats and locations sampled in both years.

Using our particulate mass loading data, glacier algae cell dimensions^14^, the algal cell-nutrient content calculation in Montagnes, et al.^22^, and accounting for the abundance of heterotrophic bacteria the Dark Zone^23^, we have calculated that the TOC and TN measured in our H_bio_ ice samples translate to glacier algal cell concentrations of 1.2–5.6 × 10^4^ cells mL^−1^ (see Supplementary Note 2 for calculation details). These values are comparable to glacier algal cell abundances reported in the area^11,15,24^ and consistent with the average glacier algal cell abundance of 2.9 ± 2.0 × 10^4^ cell mL^−1^ we reported in Cook, et al.^16^ for the same region in 2016. Note, our calculated cell counts may be a slight overestimation due to some of the measured TOC being present in the form of microbial exopolymer or dead cells. This calculation does, however, reveal that <1% of the total TOC is present in the form of bacteria, thereby confirming that the majority of the solid-phase nutrients measured on the ice surface are found in glacier algae.

The measured P_min_ is likely sourced from trace hydroxylapatite [Ca_5_(PO_4_)_3_(OH); < 1.1 wt%], which we have identified in Dark Zone surface ice dust using Rietveld refinement^25^ of powder X-ray diffraction (XRD) data (Supplementary Table 6). The mineralogy of the dust was dominated by plagioclase feldspars (41−54 wt%) and quartz (18−30 wt%) (Fig. 3e, Supplementary Note 3, Supplementary Fig. 3, Supplementary Tables 6 and 7). Ferromagnesian phases including amphiboles (4−14 wt%) and pyroxenes (<10 wt%), along with potassium feldspars (3−12 wt%), micas (1−6 wt%), and kaolinite (<3 wt%) comprise the remaining fraction.

### Nutrient biomining by supraglacial microbes

The hydroxylapatite presents an important link between mineral dust and glacier algal blooms because it contains bio-essential phosphorus^26^. Mineral abundances and meltwater chemistry together suggest increased mineral weathering with proximity to the ice sheet margin; H_bio_ ice closer to the margin (Sites 4 and 5) contained lower abundances of ferromagnesian phases than inland sites, with the corresponding meltwater enriched in dissolved major cations (Na^+^, Mg^2+^, Al^3+^, K^+^, Ca^2+^, Fe^2+^) (Fig. 3f, Supplementary Note 4, Supplementary Table 8). Elevated concentrations of soluble cations in H_bio_ ice may be due to increased rates of abiotic or biotic (heterotrophic bacteria or fungi) mineral dust weathering, or through retention of cations via adsorption to negatively charged cell exteriors.

**Fig. 4:**
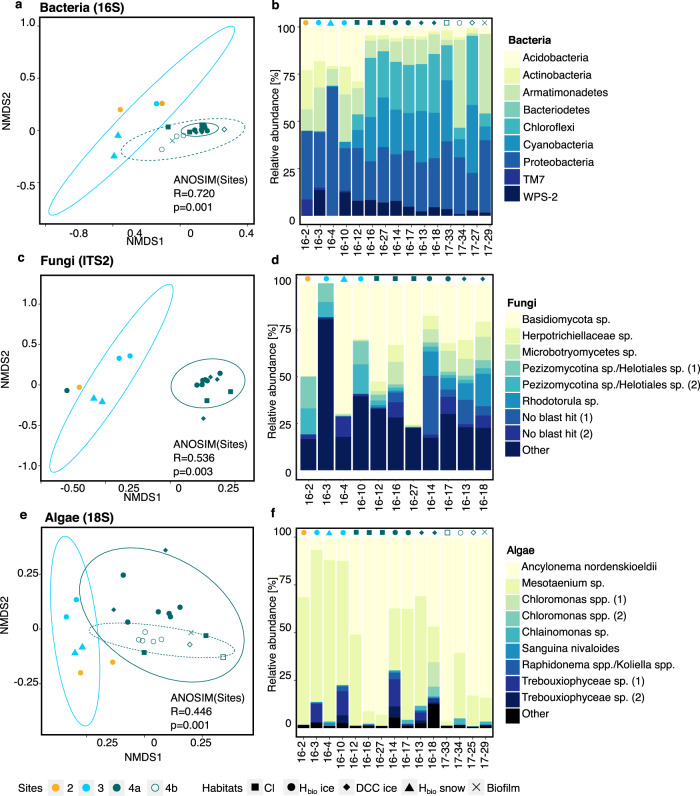
Composition of bacterial, fungal, and algal communities in surface habitats across the Dark Zone. NMDS plots showing the sample similarities for bacteria (**a**), fungi (**c**), and algae (**e**) and their respective community compositions based on relative abundances (**b**, **d**, **f**). Sites are represented by colors and habitats by point shapes. Samples cluster according to sites and dashed lines represent the 95% confidence interval. All samples with a sufficiently high number of sequences were used for the NMDS plots, whereas representative samples across sites and habitats were selected for the bar charts (details in Supplementary Tables 9–13).

**Fig. 5:**
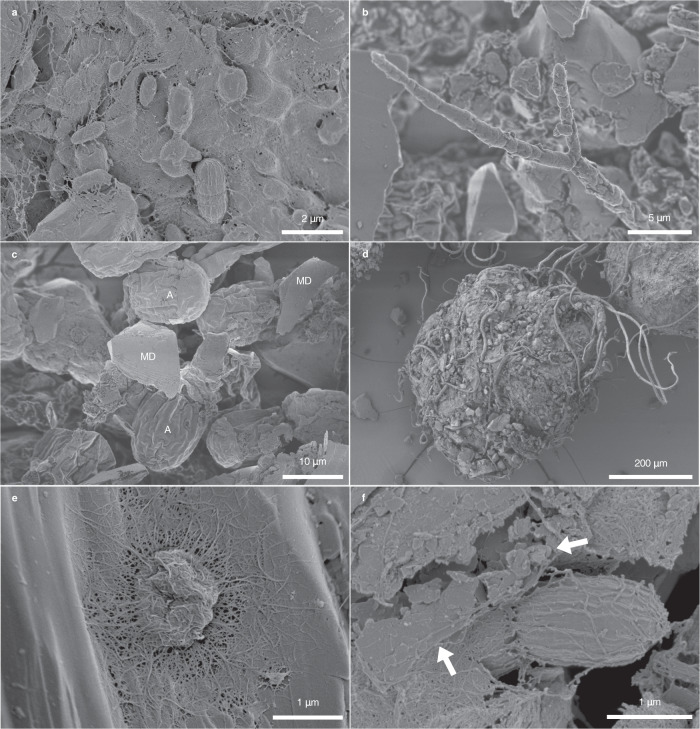
Scanning electron micrographs of cell-mineral associations in Dark Zone surface habitats. Scanning electron microscopy (SEM) micrographs showing **a** bacteria, **b** fungi, and **c** glacier algae that comprise the Dark Zone microbial community. Microbes in **c**) H_bio_ ice are more disseminated than those in **d** cryoconite granules. In all surface ice habitats, exopolymer enables microbial cells to **e** adhere to mineral surfaces, and **f** trap and bind mineral grains (arrows). In **c** A: algal cells, MD: mineral dust. Images in **a**–**f** are representative of the *n* = 12 samples observed using SEM.

Examining the composition of the microbial communities along the transect revealed that the algal (18 S), bacterial (16 S), and fungal (ITS2) community compositions clustered according to sampling sites but exhibited spatial variability across the Dark Zone (Fig. 4, Supplementary Tables 9–13). All communities showed a higher similarity within one site than within one habitat (Supplementary Note 5), indicating an association between the local geochemistry and microbiology. This is valid across sampling years; 2016 and 2017 samples collected from Site 4 were more similar than samples collected over three weeks in 2016 from Sites 2, 3, and 4a (Fig. 4). Since *Ancylonema nordenskioeldii* and *Mesotaenium* sp. comprised between 66 and 99% of the algal community in all samples (Supplementary Table 13), the assemblage of glacier algae used in the nutrient addition experiments at site 4 was reasonably representative of the region. Collectively, the microbial community (Fig. 5a–c) was intermixed with mineral dust and occurred as disseminated particulates in H_bio_ ice (Fig. 5c) and aggregated cryoconite granules in DCC ice and cryoconite hole material (Fig. 5d). Microbial exopolymer enables cells to both adhere to mineral dust (Fig. 5e) and trap and bind mineral grains (Fig. 5f).

Heterotrophic bacteria^26^ and fungi^27^ can accelerate apatite weathering beyond abiotic rates, thereby transferring solid-phase P to the organic reservoir. This accounts for the lower P_min_ concentration at sites hosting more prolific algal blooms (4 and 5), where a higher proportion of P_min_ has been transformed into P_org_ through bioweathering and algal biomass accumulation. Glacier algal productivity outstrips that of associated heterotrophic assemblages^23^, and likely drives recycling of solubilized P. Site 4a H_bio_ and DCC ice contained three to four times more dissolved P than clean ice or supraglacial stream water (Supplementary Table 8), substantiating claims that P is retained in surface ice habitats^19^.

Microbes may similarly be utilizing mineral iron; ferromagnesian minerals are less abundant in H_bio_ ice than other habitats, and decrease in abundance among H_bio_ samples by up to 60 % at sites hosting more prolific algal blooms (Fig. 3e). Dissolved iron concentrations in H_bio_ ice show a concomitant inverse trend (Fig. 3f, Supplementary Table 8). Specifically, at Site 4a, the concentration of iron was two and four-time higher in H_bio_ ice (69 ± 20 μg L^−1^) than DCC ice (36 ± 5 μg L^−1^) and clean ice (16 ± 13 μg L^−1^), respectively (Supplementary Table 8). If iron is extracted as a micronutrient, this has downstream implications for export of bioavailable iron from the ice sheet to the marine system^28^.

### Mineral dust source and transport

**Fig. 6:**
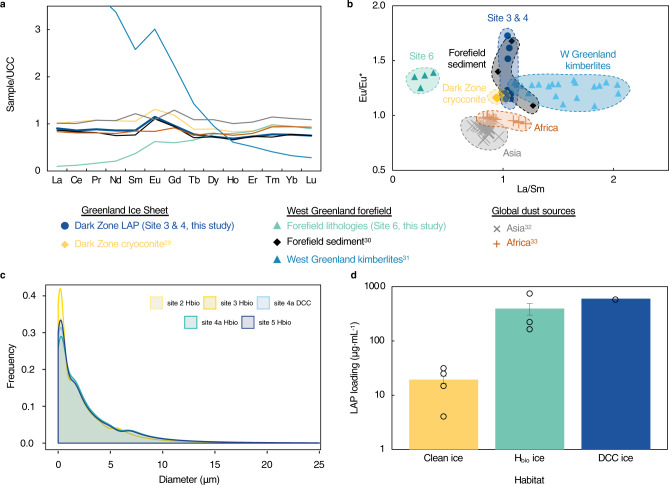
Rare Earth element signature and grain size distribution for mineral dust in Dark Zone particulates. **a**, **b** Rare Earth element (REE) normalized to the upper continental crust (UCC) compared to potential sources; **c** mineral dust size distribution; and **d** particulate mass loading by habitat. In **d** plot shows mean ± SE, clean ice: *n* = 4 samples, high algal biomass ice (H_bio_) ice: *n* = 3 samples, dispersed cryoconite (DCC) ice: *n* = 1 sample.

Our findings indicate that mineral dust facilitates glacier algal bloom development by supplying the needed P to the supraglacial algal communities. The REE (rare earth element) signatures of the mineral fraction in our surface samples were used to assess dust source. The REE signatures were homogenous and exhibited a positive Eu/Eu* anomaly analogous to local sources (Supplementary Note 6, Supplementary Table 14)^29–31^ thereby excluding distal sources characterized by a negative Eu/Eu* anomaly (Asian^32^ and African^33^ dust) as significant contributors (Fig. 6a, b). A local mineral source means that the hydroxylapatite in our particulates was derived from local apatite-bearing lithologies^34^. Local delivery of mineral dust to the GrIS is consistent with ice core records that indicate delivery of Greenlandic dust to the ice sheet during interglacial periods^35^. Furthermore, analysis of the grain-size distribution of our particulate dust fractions revealed that 99% of all grains were <20 μm in diameter (Fig. 6c), consistent with atmospheric transport processes that are typically limited to mobilizing grains <20 μm^36^.

### Mineral dust is a second-order control on Dark Zone ice albedo

We documented that H_bio_ ice contained >30× more particulate mass per volume of ice than clean ice (394 ± 194 μg mL^−1^ vs 19 ± 6 μg mL^-1^; Fig. 6d). Mineral dust accounted for 94.2 ± 0.5 wt% of these particulates (dry mass) in H_bio_ and DCC ice, with the organic matter comprising the remaining fraction (Fig. 3e, Supplementary Table 5). In spite of its dominance by mass, mineral dust is not the primary cause of ice surface darkening in the GrIS Dark Zone. In situ spectral reflectance measurements at Site 4 combined with refractive index and mineral dust grain-size distribution measurements in a radiative transfer model indicate that mineral dust has a negligible effect on albedo reduction compared to pigmented glacier algae^16^. Rather, our findings indicate that the presence of P_min_ may have a second-order effect on albedo. If this is the case, it follows that the spatial extent and melt rate of P-bearing ice may in part constrain the spatial distribution of the algal blooms producing the darkening observed on the landscape-scale.

The limited first order effect of minerals on albedo is likely due to dark-colored ferromagnesian phases (7−21 wt%) being intermixed with felsic phases (79−93 wt%), namely feldspars and quartz, which are adept at scattering light. The measured refractive index of the dust^16^ indicates that light scattering by felsic mineral grains supersedes light absorption by their ferromagnesian counterparts. Note, these findings may not apply to all locations; mineral dust can lower snow and ice albedo in glacier^37^ and ice sheet environments^38,39^. This is dependent on the bulk complex refractive index of the dust, which is a product of dust composition and grainsize. This is compounded by factors such as the distribution of dust within the ice matrix, mass mixing ratio, ice grain size and shape, and ambient illumination conditions^40^. The high proportion of felsic mineral grains may also indirectly contribute to albedo reduction via lensing of pigmented algal cells in the same manner that mineral grains enhance light absorption by black carbon nanoparticles through lensing^41^. This effect depends on the structure and mixing ratio of heterogeneous LAP aggregates in snow and ice. Its potential to contribute to ice sheet albedo reduction remains unexplored.

### Microbes, minerals, and melting: a positive feedback system

Previous studies have made links between snow algae and mineral derived nutrients^42–44^, demonstrated that snow algae respond to the addition of fertilizer^45^, and inferred that glacier algal abundance correlate with mineral dust loading^24^. Here we demonstrate how glacier algae respond in situ to the addition of specific nutrients and link this response to collocated nutrient-bearing mineral dust. Our findings demonstrate that mineral nutrient availability is a second-order control on albedo by modulating glacier algal bloom development. Comparable datasets spanning the GrIS are therefore required to incorporate mineral dust as a factor in the positive feedback between algal growth and surface melting. Algae induced melting liberates ice-bound dust, from which heterotrophs can extract micronutrients. Recycled nutrients augment glacier algal blooms, thereby further reducing albedo and promoting additional melting. Furthermore, trapping and binding of mineral dust by microbial exopolymers (Fig. 5f), helps retain valuable nutrients in the ice habitat. Notably, the glacier algal biofilm contained the highest abundance of hydroxylapatite of all samples (1.1 wt%; Supplementary Table 6), suggesting preferential entrapment of hydroxylapatite in these complex microbial colonies. Microbial retention of mineral dust reinforces the biological-albedo reducing feedback by prolonging colocation of algae and essential nutrients on the ice surface.

Nevertheless, factors controlling the timing, spatial extent, and intensity of algal blooms remain knowledge gaps limiting our ability to project biological albedo reduction and melt. Bloom initiation depends on bare ice exposure following snowpack retreat, as indicated by glacier algal cell counts for the same locations^9^, and the fact that growth of cryophilic algae can be stimulated by the presence of liquid water^45^. It is essential to constrain the timing of bloom development following snowpack retreat because satellite imagery indicates that surface darkening occurs within days of snow clearance^7^. Since 2000, the melt season in the GrIS Dark Zone has progressively started earlier, lasted longer, and exhibited greater albedo reduction^7,46^. Years experiencing earlier winter snowpack retreat yield more expansive algal blooms^47^. The higher P_min_ measured at inland sites (2 and 3) indicate that these locations are geochemically primed to host future glacier algal blooms. These trends may be exacerbated by increased atmospheric delivery of mineral dust to the ice sheet through increased windblown dust from exposed forefield lithologies^48^, and by projected increased snowfall over the GrIS^49^.

The complexity of this rapidly changing Arctic system makes it difficult to anticipate future changes to ice sheet albedo, melt rates, and contributions to sea level. Our data provide a quantitative link between mineral-derived nutrients and glacier algae blooms, and demonstrate that mineral dust is an essential nutrient source for glacier algae. This biogeochemical process must therefore be incorporated into predictive models thereby improving our understanding of how glacier algal blooms will contribute to ice sheet melting in the future.

## Methods

### Sample collection and processing

Surface snow and ice samples were collected along a transect across the ablation zone of the southwestern margin of the Greenland Ice Sheet during the 2016 (July 27–August 17) and 2017 (June 1–28) melt seasons (Fig. 1). Site 4 was the basecamp location in both seasons, differentiated as 4a (2016) and 4b (2017). Sites 1, 2, 3, and 5 were sampled only in 2016. The data presented are for samples representing a range of surface snow and ice habitats. The clean snow sample (GR16_1) collected at site 1 provides a reference for snow chemistry from the accumulation zone. The sample did not contain sufficient particulate mass for solid-phase chemical and mineralogical analyses. The collected samples were classed into the following categories based on macroscopically visible characteristics: clean snow (*n* = 1), clean surface ice (CI, *n* = 4), high algal biomass ice (H_bio_; *n* = 19), high algal biomass snow (*n* = 2), and dispersed cryoconite ice (DCC; *n* = 5). In addition, supraglacial stream water (*n* = 2), a sample of cryoconite hole (CCH) material (*n* = 1), a cryoconite hole layer from an ice core (*n* = 1), and a floating algal biofilm (*n* = 1) were collected. Details of sample types and collection locations are in Supplementary Table 1. Clean was defined as surface snow and ice containing no macroscopically visible particulates, H_bio_ ice and snow consisted of surface ice and snow containing visible glacier algal and particulate material, and DCC consisted of ice surfaces covered in disseminated particulate material from melted out cryoconite holes. Cryoconite hole material was sampled to provide a biogeochemical reference for the DCC samples. The biofilm sample consisted of a semi-coherent slick of aggregated microbial and particulate material floating on the surface of ponded meltwater. Ice and snow samples were collected from the top 3–5 cm of surface into sterile plastic bags, melted at ambient temperatures (5–10 °C) (details in^50,51^). Aliquots of filtered melted samples were processed as described below for fluid chemistry analyses. While on the ice, melted samples were filtered through glass fiber filters (GFF, pore size: 0.7 μm), from which the accumulated LAP were removed using a stainless steel spatula. The collected solid LAP were air-dried and stored in glass vials.

Three rock samples representing lithologies in the catchment area draining the west Greenland Ice Sheet were collected from outcrops near the terminus of Russell Glacier (site 6 in Fig. 1a). These samples provided a local rare Earth element signature for comparison to that of the particulate samples, as described below.

### Nutrient addition experiments

A nutrient addition incubation experiment was performed during the 2017 ablation season at our base camp location (Site 4) to determine the limiting nutrients for glacier algal assemblages. Five, 20 × 20 × 2 cm depth surface ice areas containing a conspicuous loading of glacier algae were sampled on June 22^nd^ 2017 into sterile Whirl-Pak bags, and melted in the dark for 24 h under ambient on-ice conditions (5–10 °C). These samples were used as the inoculum for the incubation experiments and had algal counts of 8.0 ± 2.1 × 10^3^ cells mL^−1^ (mean ± SD, *n* = 5). Algal cells were counted using a modified Fuchs-Rosenthal haemocytometer (Lancing, UK) on a Leica DM 2000 epifluorescence microscope with attached MC120 HD microscope camera (Leica, Germany). The inoculum was incubated in 30 mL microalgal culturing flasks (Corning, UK) in quadruplicates across five nutrient treatments: control (no nutrient addition), +NH_4_^+^ (10 µM final concentration), +NO_3_^−^ (10 µM final concentration), +PO_4_^3−^ (10 µM final concentration), and +ALL nutrients (10 µM of each NH_4_ and NO_3_; 2 µM of PO_4_ to maintain a 10:1 N:P ratio across treatments). Nutrient concentrations were designed to provide ~10-fold ambient dissolved inorganic nitrogen (DIN) and dissolved inorganic phosphorus (DIP) concentrations previously reported for GrIS supraglacial ice^52,53^.

After 24 h, 72 h, and 120 h, measurements of variable chlorophyll fluorescence were performed on 3 mL incubation sub-samples with a WaterPAM fluorometer and attached red-light emitter/detector cuvette system (Walz GmBH, Germany). During the experiment, the ambient air temperature ranged between −4 and +4 °C. Rapid light response curves (RLCs) were performed to constrain glacier algae photophysiology^17^, providing information on energy use from limiting through to saturating levels of irradiance^54^. All samples were dark-adapted for 20 minutes prior to RLC assessment, which was undertaken with a saturating pulse of ca. 8,600 μmol photons m^−2^ s^−1^ for 600 ms duration and nine 20 s incremental light steps ranging from 0 to 4000 μmol photons m^−2^ s^−1^. Maximum quantum efficiency (*F*_v_*/F*_m_) was calculated from minimum (*F*_o_) and maximum (*F*_m_) fluorescence yields in the dark-adapted state as *F*_v_*/F*_m_ = (*F*_m_ − *F*_o_)/*F*_m_. Electron transport through photosystem II (PSII) was calculated from PSII quantum efficiency (*Y*_PSII_) in relative units (*rETR* = *Y*_PSII_ × *PAR* × 0.5) assuming an equal division of photosynthetically active radiation (PAR) between photosystem I and PSII. Analysis of rapid light curves (*rETR* ~ PAR) followed^17^ with iterative curve fitting in R (v.3.6.0) and calculation of the relative maximum electron transport rate (*rETR*_max_), theoretical maximum light utilization coefficient (*α*), and light saturation coefficient (*E*_k_) following Eilers and Peeters^55^. Statistical differences in photophysiological parameters were assessed using two-way ANOVA with the fixed variables of treatment (5 levels) and date (2 levels: 24 and 120 h) and the interaction term, following tests of homogeneity of variance and normality of distribution. Tukey HSD tests were used to assess statistically significant differences in quantum efficiency and relative maximum electron transport rate between nutrient treatments. Simultaneous to photophysiological measurements, a further 5 mL of homogenized sample was fixed using 25% glutaraldehyde at 2% final concentration and transported back to the University of Bristol, UK, to assess glacier algal cell abundance (cells ml^−1^)^9^, completed within 1 month of sample return. Detailed statistical outputs and final cell count data can be found in Supplementary Tables 2–4).

### Phosphorus extractions

The phosphorus content of the H_bio_ ice, DCC ice, H_bio_ snow, and CCH samples was determined using a modified version of the SEDEX sequential extraction protocol^20^. Steps I, III, IV, and V were completed as a means of quantifying loosely bound/exchangeable P (P_exch_), mineral P (P_min_), and organic P (P_org_). The extracted P was measured in the fluid phase as described below for the melted ice samples. Detailed results can be found in Supplementary Table 5.

### Meltwater fluid chemistry

Melted surface samples and supraglacial stream water samples were filtered using 0.22 μm single use syringe filters into acid-washed Nalgene bottles. Inductively-coupled plasma mass spectroscopy (ICP-MS; Thermo Fisher iCAPQc) was used to measure fluid phase cations in the filtered water samples that were acidified using Aristar HNO_3_. ICP-MS was conducted by Stephen Reid at the University of Leeds, UK. Phosphorus was either measured using segmented flow-injection analysis (AutoAnalyser3, Seal Analytical), or for samples containing lower concentration of phosphorus using a 100 cm WPI Liquid Waveguide Capillary Cell in conjunction with an Ocean Optics USB2000 + spectrophotometer with a precision of 1.6% and a LOD of 2 nmol L^−1^. Aliquots of the non-acidified 0.22 μm filtered samples were also analyzed in replicates by ion chromatography by Andrea Viet-Hillebrand at the German Research Centre for Geosciences, Potsdam, Germany. Analyses were carried out with a conductivity detector on a Dionex ICS 3000 system, equipped with an AS 11 HC Dionex analytical column run at 35 °C for chromatographic separation of the anions. Standards containing all investigated inorganic ions (F^−^, Cl^−^, SO_4_^2−^, NO_3_^−^, PO_4_^3−^) were analyzed and all replicate samples had a standard deviation <10%. Detailed results can be found in Supplementary Table 8.

### Carbon and nitrogen content

Aliquots from the 0.7 µm GFF filtered, dried and hand-milled samples were analyzed for their bulk total and organic carbon (TC and TOC) and total nitrogen (TN) content. This was done for the H_bio_ ice, DCC ice, H_bio_ snow, and cryoconite hole material using an elemental analyzer (NC2500 Carlo Erba, standard deviation < 0.2 %, precision 0.1 %) and with the TOC concentrations subsequently measured following an in situ decalcification step. Note, the organic carbon fraction includes a contribution from black carbon. TC/TN analyses were conducted by Birgit Plessen and Sylvia Pinkerneil at the German Research Centre for Geosciences, Potsdam, Germany. Detailed results found in Supplementary Table 5. Pearson’s product-moment correlation *r*-values in Fig. 3 and supplementary Fig. 2 were calculated using Excel (v16.xx).

### Mineralogy

The mineralogy of the dust was determined using a Bruker D8 Advance Eco X-ray diffractometer (Bruker, Billerica, USA) with a Cu source, operated at 40 kV and 40 mA at the University of Leeds, UK. Samples were hand-milled in an agate mortar and pestle prior to loading in 5 or 10 mm low-background silicon mounts. The small quantity of material per sample necessitated the use of shallow sample mounts, thereby making the sample not infinitely thick with respect to X-rays, and thus rendering this analysis semi-quantitative. Furthermore, because it was necessary to rely on hand grinding, XRD patterns exhibit the effects of non-ideal particle size statistics and preferred orientation on some phases, which can result in higher R_wp_ values. The 2016 COD and 1996 ICDD databases were used to complete phase identification for each sample, in conjunction with DIFFRAC^*plus*^ Eva v.2 software^56^. Topas V 4.2^56^ and the fundamental parameters approach^57^ were used to complete Rietveld refinements^25,58,59^. No preferred orientation corrections were used because refinements are typically more accurate for samples containing many phases that are known to exhibit severe preferred orientation (e.g., phyllosilicates) when such corrections are excluded^60^. In some cases, the use of multiple K-feldspar, plagioclase feldspar, and orthopyroxene structures were used in a single refinement because this approach provided substantially improved fit statistics and visual fits to observed XRD patterns. This may reflect the incorporation of dust from multiple source rocks of differing mineralogical composition. Mineral phases identified using XRD were grouped into the following classes: quartz, plagioclase feldspars (albite/andesine/anorthite), amphiboles (refined using the structure of actinolite), potassium feldspars (orthoclase/microcline), pyroxene (enstatite/augite/diopside), and micas (refined using the structure of muscovite). Detailed results are found in Supplementary Tables 6 and 7.

### Microbial community composition

A total of 26 samples comprising 15 high algal biomass ice (H_bio_ ice), two high algal biomass snow (Hbio_snow), one biofilm, four dispersed cryoconite (DCC) (macroscopically visible particles), and four clean ice (CI) samples (without macroscopically visible particles) were collected into sterile 50 mL centrifuge tubes (H_bio_ ice, H_bio_ snow, DCC, Biofilm) or sterile sampling bags (CI). After gentle thawing at field-lab temperatures (~5–10 °C), and concentrating by gravimetric settling of particles (for H_bio_ ice, H_bio_ snow, DCC, Biofilm) or filtering (CI) through sterile Nalgene single-use filtration units (pore size 0.22 µm), up to 5 replicate from the concentrates or 1 filter per sampling event were transferred to 5 ml cryo-tubes and immediately frozen in liquid nitrogen. Samples were returned to the German Research Centre for Geosciences in Potsdam, Germany in a cryo-shipper at liquid nitrogen temperatures and stored at −80 °C until processing.

DNA was extracted from all samples using the PowerSoil (H_bio_ ice, H_bio_ snow, DCC, Biofilm) or PowerWater (CI) DNA Isolation kits (MoBio Laboratories). The 16 S rRNA, 18 S rRNA and ITS amplicons were prepared according to the Illumina “16 S Metagenomic Sequencing Library Preparation” guide. 16 S rRNA genes were amplified using the bacterial primers 341 F (5′-CCTACGGGNGGCWGCAG) and 785 R (5′-GACTACHVGGGTATCTAATCC) spanning the V3-V4 hypervariable regions. 18 S rRNA genes were amplified using the eukaryotic primers 528 F (5′ GCGGTAATTCCAGCTCCAA) and 706 R (5’ AATCCRAGAATTTCACCTCT; Cheung et al., 2010) spanning the V4-V5 hypervariable regions. ITS amplicons were amplified using the primers 5.8SbF (5′ CGATGAAGAACGCAGCG) and ITS4R (5′ TCCTCCGCTTATTGATATGC) spanning the ITS2 region. All primers were tagged with the Illumina adapter sequences. Polymerase chain reactions (PCR) were performed using KAPA HiFi HotStart ReadyMix. Initial denaturation at 95 °C for 3 min was followed by 25 cycles of denaturation at 95 °C for 30 s, annealing at 55 °C for 30 s, and elongation at 72 °C for 30 s. The final elongation was at 72 °C for 5 min. All PCRs were carried out in reaction volumes of 25 µL. All pre-amplification steps were done in a laminar flow hood with DNA-free certified plastic ware and filter tips. Amplicons were barcoded using the Nextera XT Index kit. The pooled library was sequenced on the Illumina MiSeq using paired 300-bp reads at the University of Bristol Genomics Facility, Bristol, UK.

The sequenced 16 S, 18 S, and ITS2 libraries were individually imported into Qiime2 (v.2019.1)^61^. Itsxpress was used to extract the precise ITS2 region, and thus removing the conserved regions, from the ITS2 libraries before further processing (--p-region ITS2, _--p-taxa ALL). The imported libraries were quality-filtered using the dada2 pipeline (16 S: --p-trunc-len-f = 280, --p-trunc-len-r = 200, --p-trim-feft-f = 10, --p-trim-left-r = 10; 18 S: --p-trunc-len-f = 250, --p-trunc-len-r = 200, --p-trim-feft-f = 10, --p-trim-left-r = 10; ITS2: --p-trunc-len-f = 0, --p-trunc-len-r = 0, --p-trim-feft-f = 0, --p-trim-left-r = 0). The amplicon sequence variants (ASV) in the filtered libraries were classified using classify-sklearn and the respective databases Greengenes (16 S, “gg-13-8-99-nb-classifier”)^62^, Silva (18 S, “silva-132-99-nb-classifier”)^63^, and Unite (ITS2, “unite_ver8_99_02.02.2019”)^64^. ASVs skewing the results were removed from each data set (16 S: --p-exclude Chloroplast, mitochondria; 18 S: --p-exclude Archaea, Bacteria). Feature tables containing solely algal (18 S: --p-include Chloroplastida, Ochrophyta) or fungal (ITS2: --p-include Fungi) sequences were created. Subsequently, all feature tables were rarefied to the lowest yet sufficient sample size and low-coverage samples below this threshold were discarded (16 S: 5500, ITS2: 15000, 18 S: 15000). Further, only ASVs with a minimum frequency count of 10 were retained in the feature tables. Detailed results can be found in Supplementary Tables 9–13.

The filtered feature tables were imported into R (v.3.6.0) to create bar charts representing the respective community compositions based on their relative abundances. Non-metric multidimensional scaling (NMDS) analyses were performed using the “metaMDS” function (Bray-Curtis distances) of the R package “vegan” and plots were created using the package “ggplot2”. Analysis of similarities (ANOSIM) was carried out using the “anosim” function of the “vegan” package and “sites” and “habitats” as treatment groups.

### Scanning electron microscopy (SEM)

H_bio_ ice, cryoconite, DCC ice, and biofilm samples were fixed using 2.5 % v/v glutaraldehyde and stored at 4 °C. Samples were dehydrated via an ethanol dehydration series (25%, 50%, 75%, 100%, 100%, 100%) for 15 min at each step, followed by 10 min in each of: 50:50 ethanol: hexamethyldisilazane (HMDS), and 2 × 100% HMDS. The HMDS was removed and the samples were air-dried prior to being mounted on stainless steel stubs using adhesive carbon tabs. Samples were coated with 5 nm of iridium using an Agar High Resolution sputter coater. SEM characterization of the samples was conducted using a Hitatchi 8230 SEM at the Leeds Electron Microscopy and Spectroscopy Centre (LEMAS), University of Leeds, UK.

### Rare Earth Element analysis and data compilation

Rare Earth Element (REE) analysis was conducted on H_bio_ ice, DCC ice, and cryoconite hole particulate solid materials filtered onto 0.7 µm GFF and hand-milled in an agate mortar and pestle. To remove organic matter, samples were ashed in a slightly open ceramic crucible in a muffle furnace at 450 °C for 4 h. Rock samples collected from site 6 by Gilda Varliero and Gary Barker (University of Bristol, UK) (representing lithologies of the catchment area) were cut into centimeter-sized cubes prior to milling in a tungsten ring mill. Acid dissolution of the mineral fraction was achieved in Savillex® beakers using pro-analysis acids previously purified by distillation and sub-boiling. Dissolution was first performed with 2 mL 14 M HNO_3_ and 1 mL 23 M HF on a hot plate at 120 °C for 48 h and later, after evaporation to dryness, with 2 mL 6 M HCl on a hot plate at 120 °C for 24 h. REE concentrations were determined using HR-ICP-MS (ThermoFisher Element 2) at the Vrije Universiteit Brussel, Belgium. Trace element concentrations were calibrated using elemental standard solutions and USGS reference material (AGV-2). Precision for all elements is better than 2% RSD. Detailed results can be found in Supplementary Table 14.

### Mineral dust particle size distribution analysis

Approximately 100 mg of each sample was transferred to a 50 mL centrifuge tube, to which 35 mL of 30% H_2_O_2_ (w/w) (Honeywell Fluka™) was added in order to remove the organic content. The tubes were sonicated (VWR ultrasonic cleaner) for 10 min to disaggregate the solids. The samples were agitated in an orbital shaking incubator operating at 100 rpm at 35 °C. After 72 h, the samples were centrifuged at 4000 rpm for 10 min (Eppendorf centrifuge 5810). The supernatant was removed and replaced with new H_2_O_2_. This was repeated six times until no more organic oxidation was observed. The mineral fraction was washed three times in water (Sartorius arium pro ultrapure water) for 24 h, with centrifugation succeeding each wash. The organic-free mineral fractions were dried at 35 °C prior to particle size analysis measured by Kerstin Jurkschat using a DC24000 CPS disc centrifuge^65^ at Oxford Materials Characterisation Services, Oxford, UK.

### LAP mass loading quantification

Aliquots of melted snow and ice samples of known volumes were filtered in the field successively through pre-weighed 5 μm and 0.2 μm polycarbonate filters. The filters were returned to the University of Leeds, UK where they were dried and weighed to determine the mass of LAP per volume of melted sample. The sum of the total organic carbon and nitrogen was used as a proxy to indicate the biomass fraction of each sample, with the remaining sample mass allocated to mineral dust. These values were used to calculate the mineral dust mass loading per unit of melted ice.

### Reporting summary

Further information on research design is available in the Nature Research Reporting Summary linked to this article.

## Supplementary information

Supplementary Information

Reporting Summary

## Data Availability

Detailed microbial community, and fluid and solid phase chemistry results are available in the supplementary information file. The microbial community data is available through the sequence read archive under accession number PRJNA564214. The COD database is available here: http://www.crystallography.net/cod/. Figures that have associated raw data: 2,3,4,6.

## Code availability

Code for processing the rapid light curves (RLC) was produced by C Williamson and is available here: https://github.com/chrisjw18/rlcs
