# Supplementary material for: Mineral phosphorus drives glacier algal blooms on the Greenland Ice Sheet
Source: Nat Commun. 2021 Jan 25;12:570. doi: 10.1038/s41467-020-20627-w (PMC7835244; doi:10.1038/s41467-020-20627-w)
Supplement: Supplementary file 1 — Supplementary Information [file 41467_2020_20627_MOESM1_ESM.pdf]

Supplementary Information for

MINERAL PHOSPHORUS DRIVES GLACIER ALGAL BLOOMS ON THE GREENLAND ICE SHEET

by McCUTCHEON *et al.*

Supplementary Notes 1 to 6
Supplementary Figures 1 to 3
Supplementary Tables 1 to 14
Supplementary References

Supplementary Notes

Supplementary Note 1

It should be noted that while algal abundances increase with proximity to the ice sheet margin, our TOC data appear to demonstrate the opposite trend, with LAP TOC concentrations at sites 4 and 5 being slightly lower than those at sites 2 and 3. This discrepancy is likely a consequence of how algal counts (per unit volume of melted ice) and TOC (per unit mass of solids) were quantified, with the latter being dependent on the mass of mineral dust present at the ice surface. Sites 4 and 5 are within the spatial extent of the region of outcropping mineral dust (*25*), which may explain the slightly suppressed TOC values. The ratio of TOC:N:P_{org} measured in the H_{bio} LAP samples collected at Site 4 ranged between 690:48:1 and 2615:196:1.

Supplementary Note 2

Total particulate mass loading for H_{bio} ice (394 ± 336 $\mu g \cdot mL^{-1}$, mean ± SD, n=3) was used to calculate glacier algal cell abundance from the measured TOC and TN data (Supplementary Table 5). These values were used to calculate the average quantity of TOC (20.5 $\mu g \cdot mL^{-1}$) and TN (0.8 $\mu g \cdot mL^{-1}$) per volume of melted H_{bio} ice sample. Glacier algal cell dimensions (29.60 ± 7.61 µm × 12.04 ±2.06 µm[1]) were used to calculate average cell volume (3370 μm^3). This cell volume was used along with the TOC and TN per volume and the cell-nutrient conversion from Montagnes, et al. [2] to calculate glacier algal cells per mL. This calculation generated values of 6.01×10^4 and 1.18×10^4 $cells \cdot mL^{-1}$ from TOC and TN respectively.

The glacier algal cell estimate from TOC was improved by accounting for the presence of black carbon and heterotrophic bacteria. From unpublished data, we know that black carbon accounts very little of the measured TOC, and not more than 5% of measured TOC in H_{bio} ice samples. The TOC value per mL was therefore reduced by 5%. The average

heterotrophic bacteria cell abundance at Site 4 has been reported as $3.3 \pm 0.3 \times 10^5$ cell·mL^{-1} [13]. Using a hypothetical bacterial cell volume of 1 μm^3 and the bacteria-carbon calculation in Bratbak and Dundas [4], we calculate that bacteria account for 7.62×10^{-2} μg C·mL^{-1}, or 0.4% of the total TOC. After removing this quantity of carbon from the TOC the glacier algal cell estimate from TOC was recalculated to be 5.63×10^4 cell·mL^{-1}. The range of values reported in the main text (1.2–5.6 × 10^4 cell·mL^{-1}) refer to the estimates from TN and the adjusted TOC.

Supplementary Note 3

Little to no inorganic carbon was detected in the samples through the TC/TOC analyses (Supplementary Table 5), suggesting the absence of carbonate minerals, a result that was corroborated by XRD (Supplementary Table 6). Low abundances of smectite phases detected in some samples were excluded from the quantitative refinement (Supplementary Table 6). H_{bio} ice dust contained higher abundances of framework silicates and lower abundances of single, double and phyllosilicate phases compared to DCC ice dust. The composition of dust in H_{bio} ice from site 4 in 2016 and 2017 were similar, with the exception that the 2017 H_{bio} ice contained greater abundances of single chain silicates (pyroxene) and phyllosilicate phases (Supplementary Fig. 3a, Supplementary Table 7). The dust composition in H_{bio} ice exhibited a spatial trend across the ablation zone, which along with the corresponding meltwater fluid chemistry, provides an indication of mineral weathering processes. H_{bio} ice closer to the margin (Site 5) was enriched in framework silicates (90 wt%) and depleted in phyllo-, single chain, and double chain silicate phases (10 wt%) compared to inland H_{bio} ice (Site 2: 71 wt% and 29 wt%, respectively) (Supplementary Fig. 3a, details and statistics: Supplementary Table 7).

Supplementary Note 4

By habitat, cation concentrations were higher in the 2016 samples (site 4a) than their 2017 (site 4b) counterparts, likely due to them being collected later in the melt season (Supplementary Fig. 3b). The 2017 samples contained higher concentrations of chloride than those collected in 2016 (Supplementary Table 6). Specifically, H_{bio} and DCC ice at Site 4 contained higher concentrations of ions in solution than clean ice and supraglacial stream water, an occurrence that may be the product of mineral weathering by heterotrophic bacteria or fungi (Supplementary Fig. 3b). Solid phase P_{org} concentrations did not correlate with P_{exch} or aqueous P (Supplementary Fig. 2d,e).

Supplementary Note 5

All communities showed a significantly higher similarity within one site (16S: R=0.720, p=0.001; 18S: R=0.446, p=0.001; ITS2: R=0.536, p=0.003) than within one habitat (16S: R=0.170, p=0.144; 18S: R=0.303, p=0.012; ITS2: R=0.207; p=0.102).

Supplementary Note 6

All REE data were normalized (n) to the average elemental composition of the upper continental crust (UCC) given by Rudnick and Gao [5]. Europium anomaly was calculated as follows:

$$\mathrm{Eu/Eu^{*}} = \mathrm{Eu_n}/\sqrt{\mathrm{Sm_n} \times \mathrm{Gd_n}}$$

H_{bio}, DCC, and CCH REE patterns exhibit preeminent positive Eu*/Eu anomaly that make them distinct from distal sources such as Asian dusts from Northern China and the Taklamakan Desert[6] as well as African[7] dusts collected in the North Atlantic (Fig 6b). H_{bio}, DCC, and CCH REE patterns correspond well with the REE signature of local sediments. Our LAP samples can be subdivided into two groups based on their concentration and pattern shape, thereby matching: (i) the signature of Qinnguata Kuusua river sediment (the river that drains Kangerlussuaq fjord), and (ii) the average of local sediments in the catchment area[8]. Our REE results are consistent with the average REE signature measured in cryoconites

collected from a location near our sampling transect[9]. The rock samples collected in the Russel glacier forefeels, however, plot separately. This may indicate that rock weathering and diagenetic processes altered the REE patterns. Overall, our REE results indicate that local sources are the dominant input of the mineral dust in our LAP samples, and also demonstrate the variability in REE signature observed in local sediments and lithologies.

Supplementary Tables

Supplementary Table 1. Locations, dates and sample types collected for particulate analyses. Sites 4a and 4b were the base camp locations for 2016 and 2017, respectively.

Site	Distance to margin (km)	Location	Date sampled	Sample ID	Sample Type
1	130	N67.0006 W47.02575	2016-07-27	GrIS16_1	Clean snow
2	75	N67.28250 W48.68000	2016-07-27	GrIS16_2	H_{bio} ice
		N67.191389 W48.68250	2016-08-05	GrIS16_9	H_{bio} ice
3	50	N67.19222 W49.08000	2016-07-27	GrIS16_3	H_{bio} ice
		N67.21583 W49.04250	2016-07-27	GrIS16_4	H_{bio} snow
		N67.21861 W49.15667	2016-08-05	GrIS16_10	H_{bio} ice
		N67.22889 W49.12306	2016-08-05	GrIS16_11	H_{bio} snow
4a	35	N67.195 W49.42056	2016-07-31	GrIS16_5	H_{bio} ice
		N67.19722 W49.42917	2016-07-31	GrIS16_6	H_{bio} ice
		N67.195 W49.42056	2016-08-01	GrIS16_7	H_{bio} ice
		N67.19722 W49.429167	2016-08-01	GrIS16_8	H_{bio} ice
		N67.26639 W49.35694	2016-07-30	GrIS16_12	Clean ice
		N67.26333 W49.35611	2016-07-30	GrIS16_13	DCC ice
		N67.26028 W49.35278	2016-07-30	GrIS16_14	H_{bio} ice
		N67.1975 W49.47694	2016-08-01	GrIS16_15	Stream water
		N67.23778 W49.46861	2016-08-02	GrIS16_16	Clean ice
		N67.23778 W49.46861	2016-08-02	GrIS16_17	H_{bio} ice
		N67.23778 W49.46861	2016-08-02	GrIS16_18	DCC ice
		N67.1975 W49.47694	2016-08-10	GrIS16_23	Stream water
		N67.22167 W49.59639	2016-08-14	GrIS16_27	Clean ice
		N67.22167 W49.59639	2016-08-14	GrIS16_28	H_{bio} ice
		N67.22167 W49.60028	2016-08-14	GrIS16_29	DCC ice
		N67.23861 W49.51139	2016-08-17	GrIS16_30	DCC ice
4b	35	N67.07822 W49.34330	2017-06-23	GrIS17_24	H_{bio} ice
		N67.07601, W49.34902	2017-06-23	GrIS17_25	DCC ice
		N67.076392, W49.343453	2017-06-25	GrIS17_26	H_{bio} ice
		N67.07515, W49.35309	2017-06-25	GrIS17_27	Cryoconite hole
		N67.076392, W49.343453	2017-06-27	GrIS17_28	H_{bio} ice
		N67.07515, W49.35309	2017-06-27	GrIS17_29	Biofilm
		N67.07817, W49.33974	2017-06-20	GRIS17_31	CCH core layer
		N67.07515, W49.35309	2017-06-26	GrIS17_33	Clean ice
		N67.076392, W49.343453	2017-06-25	GrIS17_34	H_{bio} ice
		N67.076392, W49.343453	2017-06-26	GrIS17_35	H_{bio} ice
5	33	N67.12556 W49.45500	2016-08-12	GrIS16_24	H_{bio} ice
		N67.12694 W49.46833	2016-08-12	GrIS16_25	H_{bio} ice
		N67.12444 W49.44778	2016-08-12	GrIS16_26	H_{bio} ice
6		N67.15347 W50.06095	2018-08-09	Rock_J1	Rock

N67.15332 W50.06577	2018-08-09	Rock_J2	Rock
N67.15372 W50.06627	2018-08-09	Rock_J3	Rock

Supplementary Table 2. Results of a Tukey HSD test with a 95% family-wise confidence interval for F_v/F_m measurements made at 24 h and 120 h in the nutrient addition experiment.

By treatment:	difference	lower	upper	adjusted p - value
Control - ALL	-0.048625	-0.120219	0.022969	0.304554
NH4 - ALL	-0.095875	-0.167469	-0.024281	0.004427
NO3 - ALL	-0.077250	-0.148844	-0.005656	0.029443
PO4 - ALL	0.002250	-0.069344	0.073844	0.999983
NH4 - Control	-0.047250	-0.118844	0.024344	0.331902
NO3 - Control	-0.028625	-0.100219	0.042969	0.773587
PO4 - Control	0.050875	-0.020719	0.122469	0.262936
NO3 - NH4	0.018625	-0.052969	0.090219	0.941443
PO4 - NH4	0.098125	0.026531	0.169719	0.003481
PO4 - NO3	0.079500	0.007906	0.151094	0.023685
By time:	difference	lower	upper	adjusted p - value
120 h - 24 h	0.083700	0.051819	0.115581	0.000008
By treatment and time:	difference	lower	upper	adjusted p - value
Control:24 h - ALL:24 h	0.020250	-0.098821	0.139321	0.999849
NH4:24 h - ALL:24 h	-0.056000	-0.175071	0.063071	0.835811
NO3:24 h - ALL:24 h	-0.046750	-0.165821	0.072321	0.935807
PO4:24 h - ALL:24 h	0.037500	-0.081571	0.156571	0.983711
ALL:120 h - ALL:24 h	0.153500	0.034429	0.272571	0.004269
Control:120 h - ALL:24 h	0.036000	-0.083071	0.155071	0.987647
NH4:120 h - ALL:24 h	0.017750	-0.101321	0.136821	0.999950
NO3:120 h - ALL:24 h	0.045750	-0.073321	0.164821	0.943315
PO4:120 h - ALL:24 h	0.120500	0.001429	0.239571	0.045490
NH4:24 h - Control:24 h	-0.076250	-0.195321	0.042821	0.487874
NO3:24 h - Control:24 h	-0.067000	-0.186071	0.052071	0.656808
PO4:24 h - Control:24 h	0.017250	-0.101821	0.136321	0.999961
ALL:120 h - Control:24 h	0.133250	0.014179	0.252321	0.018914
Control:120 h - Control:24 h	0.015750	-0.103321	0.134821	0.999982
NH4:120 h - Control:24 h	-0.002500	-0.121571	0.116571	1.000000
NO3:120 h - Control:24 h	0.025500	-0.093571	0.144571	0.999035
PO4:120 h - Control:24 h	0.100250	-0.018821	0.219321	0.158238
NO3:24 h - NH4:24 h	0.009250	-0.109821	0.128321	1.000000
PO4:24 h - NH4:24 h	0.093500	-0.025571	0.212571	0.227254
ALL:120 h - NH4:24 h	0.209500	0.090429	0.328571	0.000054
Control:120 h - NH4:24 h	0.092000	-0.027071	0.211071	0.245213
NH4:120 h - NH4:24 h	0.073750	-0.045321	0.192821	0.533121
NO3:120 h - NH4:24 h	0.101750	-0.017321	0.220821	0.145409
PO4:120 h - NH4:24 h	0.176500	0.057429	0.295571	0.000725
PO4:24 h - NO3:24 h	0.084250	-0.034821	0.203321	0.353243
ALL:120 h - NO3:24 h	0.200250	0.081179	0.319321	0.000112
Control:120 h - NO3:24 h	0.082750	-0.036321	0.201821	0.376932
NH4:120 h - NO3:24 h	0.064500	-0.054571	0.183571	0.701320
NO3:120 h - NO3:24 h	0.092500	-0.026571	0.211571	0.239118
PO4:120 h - NO3:24 h	0.167250	0.048179	0.286321	0.001489
ALL:120 h - PO4:24 h	0.116000	-0.003071	0.235071	0.061082
Control:120 h - PO4:24 h	-0.001500	-0.120571	0.117571	1.000000
NH4:120 h - PO4:24 h	-0.019750	-0.138821	0.099321	0.999878
NO3:120 h - PO4:24 h	0.008250	-0.110821	0.127321	1.000000
PO4:120 h - PO4:24 h	0.083000	-0.036071	0.202071	0.372927
Control:120 h - ALL:120 h	-0.117500	-0.236571	0.001571	0.055421
NH4:120 h - ALL:120 h	-0.135750	-0.254821	-0.016679	0.015824
NO3:120 h - ALL:120 h	-0.107750	-0.226821	0.011321	0.102284
PO4:120 h - ALL:120 h	-0.033000	-0.152071	0.086071	0.993286

NH4:120 h - Control:120 h	-0.018250	-0.137321	0.100821	0.999937
NO3:120 h - Control:120 h	0.009750	-0.109321	0.128821	1.000000
PO4:120 h - Control:120 h	0.084500	-0.034571	0.203571	0.349376
NO3:120 h - NH4:120 h	0.028000	-0.091071	0.147071	0.998010
PO4:120 h - NH4:120 h	0.102750	-0.016321	0.221821	0.137332
PO4:120 h - NO3:120 h	0.074750	-0.044321	0.193821	0.514912

Supplementary Table 3. Results of a Tukey HSD test with a 95% family-wise confidence interval for $rETR_{\max}$ measurements made at 24 h and 120 h in the nutrient addition experiment.

By treatment:	difference	lower	upper	adjusted p - value
Control - ALL	-54.187704	-96.368800	-12.006610	0.006686
NH4 - ALL	-88.229365	-130.410460	-46.048271	0.000011
NO3 - ALL	-66.073683	-108.254780	-23.892590	0.000750
PO4 - ALL	7.534635	-34.646460	49.715729	0.984855
NH4 - Control	-34.041661	-76.222750	8.139433	0.160147
NO3 - Control	-11.885979	-54.067070	30.295114	0.923211
PO4 - Control	61.722339	19.541250	103.903433	0.001695
NO3 - NH4	22.155682	-20.025410	64.336775	0.555853
PO4 - NH4	95.764000	53.582910	137.945093	0.000003
PO4 - NO3	73.608318	31.427220	115.789412	0.000179
**By time:**	difference	lower	upper	adjusted p - value
120 h - 24 h	54.464760	35.681430	73.248080	0.000002
**By treatment and time:**	difference	lower	upper	adjusted p - value
Control:24 h - ALL:24 h	-0.220758	-70.374175	69.932660	1.000000
NH4:24 h - ALL:24 h	-43.666315	-113.819732	26.487100	0.526468
NO3:24 h - ALL:24 h	-29.824681	-99.978098	40.328740	0.900679
PO4:24 h - ALL:24 h	32.396540	-37.756877	102.549960	0.849410
ALL:120 h - ALL:24 h	118.321120	48.167703	188.474540	0.000107
Control:120 h - ALL:24 h	10.166470	-59.986947	80.319890	0.999961
NH4:120 h - ALL:24 h	-14.471294	-84.624711	55.682120	0.999282
NO3:120 h - ALL:24 h	15.998435	-54.154982	86.151850	0.998427
PO4:120 h - ALL:24 h	100.993851	30.840434	171.147270	0.001078
NH4:24 h - Control:24 h	-43.445558	-113.598974	26.707860	0.533306
NO3:24 h - Control:24 h	-29.603923	-99.757340	40.549490	0.904507
PO4:24 h - Control:24 h	32.617298	-37.536119	102.770710	0.844454
ALL:120 h - Control:24 h	118.541878	48.388461	188.695290	0.000104
Control:120 h - Control:24 h	10.387228	-59.766189	80.540640	0.999953
NH4:120 h - Control:24 h	-14.250536	-84.403953	55.902880	0.999364
NO3:120 h - Control:24 h	16.219193	-53.934224	86.372610	0.998252
PO4:120 h - Control:24 h	101.214609	31.061192	171.368030	0.001047
NO3:24 h - NH4:24 h	13.841634	-56.311783	83.995050	0.999496
PO4:24 h - NH4:24 h	76.062855	5.909438	146.216270	0.025322
ALL:120 h - NH4:24 h	161.987435	91.834018	232.140850	0.000000
Control:120 h - NH4:24 h	53.832786	-16.320631	123.986200	0.253045
NH4:120 h - NH4:24 h	29.195021	-40.958395	99.348440	0.911353
NO3:120 h - NH4:24 h	59.664750	-10.488667	129.818170	0.149432
PO4:120 h - NH4:24 h	144.660166	74.506749	214.813580	0.000003
PO4:24 h - NO3:24 h	62.221221	-7.932196	132.374640	0.116253
ALL:120 h - NO3:24 h	148.145801	77.992384	218.299220	0.000002
Control:120 h - NO3:24 h	39.991151	-30.162266	110.144570	0.640908
NH4:120 h - NO3:24 h	15.353387	-54.800030	85.506800	0.998856
NO3:120 h - NO3:24 h	45.823116	-24.330301	115.976530	0.460822
PO4:120 h - NO3:24 h	130.818532	60.665115	200.971950	0.000020
ALL:120 h - PO4:24 h	85.924580	15.771163	156.078000	0.007577
Control:120 h - PO4:24 h	-22.230070	-92.383487	47.923350	0.983028
NH4:120 h - PO4:24 h	-46.867834	-117.021251	23.285580	0.430078
NO3:120 h - PO4:24 h	-16.398105	-86.551522	53.755310	0.998099
PO4:120 h - PO4:24 h	68.597311	-1.556106	138.750730	0.059409

Control:120 h - ALL:120 h	-108.154650	-178.308067	-38.001230	0.000417
NH4:120 h - ALL:120 h	-132.792414	-202.945831	-62.639000	0.000016
NO3:120 h - ALL:120 h	-102.322685	-172.476102	-32.169270	0.000904
PO4:120 h - ALL:120 h	-17.327269	-87.480686	52.826150	0.997114
NH4:120 h - Control:120 h	-24.637764	-94.791181	45.515650	0.967020
NO3:120 h - Control:120 h	5.831965	-64.321452	75.985380	1.000000
PO4:120 h - Control:120 h	90.827380	20.673964	160.980800	0.004061
NO3:120 h - NH4:120 h	30.469729	-39.683688	100.623150	0.888969
PO4:120 h - NH4:120 h	115.465145	45.311728	185.618560	0.000157
PO4:120 h - NO3:120 h	84.995416	14.841999	155.148830	0.008515

Supplementary Table 4. Glacier algal cell concentrations (cells·mL^{-1}) at the end of the 120 h nutrient incubation experiment. Glacier algae assemblage used for the incubations had an initial mean cell concentration of 8.0 ± 2.1 × 10^{3} cells·mL^{-1}.

		Control	$+NH_4^+$	$+NO_3^-$	$+PO_4^{3-}$	+ALL
**Replicate**	**1**	6.75 × 10^3	5.95 × 10^3	6.00 × 10^3	9.20 × 10^3	5.70 × 10^3
	**2**	6.95 × 10^3	5.70 × 10^3	8.05 × 10^3	6.30 × 10^3	6.55 × 10^3
	**3**	8.25 × 10^3	6.50 × 10^3	5.65 × 10^3	8.95 × 10^3	5.45 × 10^3
	**4**	6.30 × 10^3	5.95 × 10^3	5.55 × 10^3	6.00 × 10^3	8.65 × 10^3
**Mean**		7.06 × 10^3	6.03 × 10^3	6.31 × 10^3	7.61 × 10^3	6.59 × 10^3
**Standard deviation**		8.37 × 10^2	3.38 × 10^2	1.17 × 10^3	1.70 × 10^3	1.45 × 10^3
**Standard error**		4.19 × 10^2	1.69 × 10^2	5.87 × 10^2	8.48 × 10^2	7.27 × 10^2

Supplementary Table 5. Carbon, nitrogen, and phosphorus content of solid LAPs collected from melted surface ice.

			Carbon and nitrogen (wt%)				Carbon, nitrogen, and phosphorus (mol·g^{-1} of solids)					Nutrient ratios (mol·g^{-1})		
Site	Sample ID	Sample type	TC %	TOC %	TIC %	TN %	TOC mol·g^{-1}	TN mol·g^{-1}	P_{exch}	P_{min}	P_{org}	C:N	C:P	N:P
2	GrIS-16_2	H_{bio} ice	8.45	7.84	0.61	0.87	0.0065	6.21E-04	5.10E-08	2.27E-06	1.20E-05	10.5	543.4	51.7
3	GrIS-16_3	H_{bio} ice	7.19	6.99	0.20	0.72	0.0058	5.14E-04	2.97E-07	1.97E-06	1.06E-05	11.3	549.4	48.5
	GrIS-16_10	H_{bio} ice	5.61	5.62	-0.01	0.57	0.0047	4.07E-04	2.78E-07	1.10E-06	8.62E-06	11.5	542.6	47.2
	GrIS-16_11	H_{bio} snow	6.72	5.80	0.92	0.60	0.0048	4.28E-04	2.41E-06	1.29E-06	5.49E-06	11.3	879.6	78.0
4a	GrIS-16_5	H_{bio} ice	4.27	3.62	0.65	0.34	0.0030	2.43E-04	8.58E-07	2.93E-07	2.10E-06	12.4	1436.0	115.6
	GrIS-16_6	H_{bio} ice	4.54	4.30	0.24	0.40	0.0036	2.86E-04	8.96E-07	4.10E-07	3.52E-06	12.5	1017.6	81.1
	GrIS-16_13	DCC ice	8.07	7.27	0.80	0.71	0.0061	5.07E-04	3.52E-07	1.83E-06	8.60E-06	11.9	703.8	58.9
	GrIS-16_14	H_{bio} ice	2.59	2.42	0.17	0.20	0.0020	1.43E-04	6.04E-07	2.22E-07	1.85E-06	14.1	1087.1	77.0
	GrIS-16_17a	H_{bio} ice	6.44	6.69	-0.25	0.55	0.0056	3.93E-04	1.80E-07	1.39E-06	8.07E-06	14.2	690.5	48.7
	GrIS-16_17b	H_{bio} ice	6.74	6.25	0.49	0.58	0.0052	4.14E-04	2.27E-07	1.34E-06	6.96E-06	12.6	747.3	59.4
	GrIS-16_18a	DCC ice	6.36	5.80	0.56	0.54	0.0048	3.85E-04				12.5		
	GrIS-16_18b	DCC ice	6.33	6.13	0.20	0.54	0.0051	3.85E-04				13.2		
	GrIS-16_28	H_{bio} ice	3.12	3.17	-0.05	0.25	0.0026	1.78E-04				14.8		
	GrIS-16_29	DCC ice	7.48	4.78	2.70	0.62	0.0040	4.43E-04	6.58E-07	7.96E-07	5.08E-06	9.0	783.0	87.1
4b	GRIS-17-24	H_{bio} ice		8.0		0.7	0.0067	4.89E-04	1.47E-06	7.38E-07	2.55E-06	13.6	2614.9	192.1
	GRIS-17-25	DCC ice		3.7		0.4	0.0031	2.72E-04	4.41E-07	5.74E-07	3.82E-06	11.4	814.0	71.2
	GRIS-17-27	Cryoconite hole		6.6		0.6	0.0055	4.33E-04	2.31E-07	1.14E-06	8.43E-06	12.7	652.7	51.3

	GrIS-17-29	Biofilm		8.1										
	GRIS-17-34	H_{bio} ice		5.5		0.6	0.0046	4.58E-04	5.20E-07	5.88E-07	2.89E-06	10.0	1582.8	158.3
5	GrIS-16_24	H_{bio} ice	4.85	4.26	0.59	0.29	0.0035	2.07E-04	1.69E-07	2.38E-07	1.51E-06	17.1	2356.0	137.5
	GrIS-16_26	H_{bio} ice	3.69	3.10	0.59	0.24	0.0026	1.71E-04						

TC: total carbon. TOC: total organic carbon, IC: inorganic carbon, P_{exch}: exchangeable/loosely bound phosphorus, P_{min}: mineral phosphorus, P_{org}: organic phosphorus

Supplementary Table 6. Mineral phase abundances in 2016 and 2017 particulate samples as determined by Rietveld refinement with powder X-ray diffraction data. Abundances given as weight percent of total mineral dust (n=20).

Year	2016	2016	2016	2016	2016	2016	2016	2016	2016	2016	2016	2016	2017	2017	2017	2017	2017	2017	2017	2016
Sample ID	16-2	16-3	16-10	16-5	16-6	16-14	16-17	16-28	16-13c	16-18	16-29	16-30	17-24	17-26	17-28	17-34	17-35	17-27	17-29	16-24
Site	2	3	3	4a	4a	4a	4a	4a	4a	4a	4a	4a	4b	4b	4b	4b	4b	4b	4b	5
Surface habitat	H_{bio} ice	H_{bio} ice	H_{bio} ice	H_{bio} ice	H_{bio} ice	H_{bio} ice	H_{bio} ice	H_{bio} ice	DCC ice	DCC ice	DCC ice	DCC ice	H_{bio} ice	H_{bio} ice	H_{bio} ice	H_{bio} ice	DCC ice	CC Hole	Bio-film	H_{bio} ice
quartz	21.2	20.8	20.4	24.1	22.2	24.5	22.7	30.1	17.5	20.0	17.5	18.4	26.4	21.2	24.8	21.4	21.9	17.8	21.7	27.9
andesine	22.7	23.3	20.9	33.4	36.9	40.6	41.4	46.4	17.5	36.3	20.6	19.8	42.7	23.2	22.1	24.4	24.7	20.3	24.6	40.2
albite	18.2	19.5	21.6	10.2	6.2	5.8	6.7	4.7	17.7	6.3	15.9	15.2	10.6	17.8	21.9	14.6	15.9	21.5	21.4	6.2
anorthite				7.9	8.8	6.0	6.3		13.0	7.2	9.3	9.0		8.4	6.6	13.4	9.4	5.1	7.5	5.3
orthoclase	4.0	2.2	1.7	6.0	4.4	3.7	5.8	4.7	2.0	4.0	3.3	3.5	4.8	4.4	1.8	2.0	3.4			4.7
microcline	5.1	6.2	6.4	4.5	5.1	8.0	4.3	7.6	2.9	5.6	4.3	3.8	5.4	4.3	4.9	5.4	3.9	3.3	6.9	5.4
actinolite	10.5	12.0	11.7	8.7	10.1	7.7	8.0	3.9	13.0	14.2	14.5	13.4	4.1	8.1	6.2	7.2	6.4	13.0	5.1	6.1
enstatite	4.6	5.2	6.3	1.9	1.9	1.2	0.6	0.8	5.1		4.0	4.9	1.1	4.9	4.8	3.2	4.8	6.7	4.7	1.2
augite	2.0	3.8	3.9				0.9		3.8		2.9	3.4	1.1	1.3	3.3	3.4	4.2	3.2	1.7	
diopside					0.9		2.2							1.9						
muscovite	5.6	4.6	3.8	2.0	2.1	1.6	0.7	1.5	5.4	4.5	4.5	5.3	2.0	2.5	2.5	3.2	3.4	5.2	4.7	2.0
kaolinite	3.2	2.5	2.3	1.1	1.3	0.5		0.4	1.5	1.7	3.2	2.9	1.4	1.3	1.0	1.2	1.3	2.7	0.7	1.1
clinochlore	1.2																			
biotite												trace								
illite	1.7		0.8									trace						0.5		
hydroxylapatite		0.1	0.2	0.3	0.1	0.4	0.4		0.5	0.3	0.1	0.3	0.4	0.7	0.1	0.6	0.5	0.6	1.1	0.1
smectite	no	yes	yes	no	no	no	yes	yes	yes	yes	yes	yes	no	no	yes	no	no	no	no	no
Total:	100.0	100.0	100.0	100.0	100.0	100.0	100.0	100.0	100.0	100.0	100.0	100.0	100.0	100.0	100.0	100.0	100.0	100.0	100.0	100.0
R_{wp} (%)	12.4	12.3	13.4	17.0	18.6	22.2	20.6	23.4	12.2	12.9	12.1	12.0	19.5	20.3	22.6	28.6	24.3	13.0	20.8	22.7

Supplementary Table 7. Mineral class abundances in high algal biomass (H_{bio}) ice sampled across the ablation zone in 2016. Values listed in weight percent of total mineral dust % (± standard error where applicable). Two-sided t-test comparing of mineral class abundances between site 3 and 4a.

Year	Habitat	Site	n	Framework silicates	Double chain silicates	Single chain silicates	Phyllosilicates	Apatite	Total:
2016	H_{bio} ice	2	1	71.1	10.5	6.6	11.7	0.01	100.0

		3	2	71.5 ± 0.5	11.8 ± 0.1	9.6 ± 0.6	7.0 ± 0.1	0.1 ± 0.1	100.0
		4a	5	87.8 ± 1.6	7.7 ± 1.0	2.1 ± 0.5	2.2 ± 0.5	0.2 ± 0.1	100.0
		5	1	89.5	6.1	1.2	3.1	0.1	100.0
	DCC	4a	4	72.7 ± 2.2	13.8 ± 0.3	6.0 ± 2.1	7.2 ± 0.4	0.3 ± 0.1	100.0
2017	H_{bio} ice		4	83.1 ± 2.3	6.4 ± 0.9	6.3 ± 1.4	3.8 ± 0.2	0.4 ± 0.1	100.0
	DCC	4b	1	79.3	6.4	9.0	4.7	0.5	100.0
	CC Hole		1	68.1	13.0	10.0	8.4	0.6	100.0

Comparison of mineral class abundances quantified for sites 3 and 4a (Welch two sample t-test, unequal variance)

	Framework silicates	Double chain silicates	Single chain silicates	Phyllosilicates
t-value	9.6	4.0	9.5	9.8
df	4.5	4.1	2.9	4.2
p-value	0.0003	0.01	0.003	0.0005

Supplementary Table 8. Major cation and anion concentrations in the fluid phase and pH, conductivity and total dissolved solids (TDS) of supraglacial stream water and melted ice and snow samples.

			Cations (μg•L^{-1})							Anions (μg•L^{-1})						pH	Conductivity	TDS
Site	Sample ID	Habitat	Na^+	Mg^{2+}	Al^{3+}	K^+	Ca^{2+}	Mn^{2+}	Fe^{2+}	P^{3-}	F^-	Cl^-	NO_2^-	NO_3^-	SO_4^{2-}		(μS•cm^{-1})	(ppm)
1	GrIS-16_1	Clean snow	19.6	0.84	<LOD	<LOQ	<LOD	<LOQ	<LOQ	0.89	<LOD	<LOD	<LOD	<LOD	<LOD	7.64	1.86	0.51
2	GRIS-16_2	H_{bio} ice	30.18	2.41	<LOQ	<LOQ	<LOD	<LOD	24.88	0.75	<LOD	<LOD	<LOD	<LOD	<LOD	4.96	8.66	5.03
3	GrIS-16_3	H_{bio} ice	50.09	2.93	<LOQ	<LOQ	<LOD	<LOQ	15.27	0.43	<LOD	<LOD	<LOD	<LOD	<LOD	5.32	4.41	2.66
	GrIS-16_4	H_{bio} snow	44.61	16.95	5.25	<LOQ	<LOD	0.35	147.23	0.70	<LOD	972.1	<LOD	<LOD	<LOD	4.84	2.36	1.69
	GrIS-16_10	H_{bio} ice	53.51	4.44	5.03	18.55	<LOD	<LOD	17.06	0.86	<LOD	<LOD	<LOD	<LOD	<LOD	5.74	5.67	3.29
	GrIS-16_11	H_{bio} snow	35.87	1.83	<LOQ	30.65	<LOD	<LOD	19.18	1.08	<LOD	<LOD	<LOD	<LOD	<LOD	5.69	6.91	3.85
4a	GrIS-16_5	H_{bio} ice	59.77	9.48	5.74	42.89	<LOD	0.454	118.68	0.83	<LOD	<LOD	<LOD	<LOD	<LOD	6.39	1.62	1.27
	GrIS-16_6	H_{bio} ice	49.58	7.61	9.12	22.17	<LOD	0.104	34.72	0.64	<LOD	<LOD	<LOD	<LOD	<LOD	6.41	1.66	1.30
	GrIS-16_14	H_{bio} ice	43.12	12.47	5.49	25.31	<LOD	0.069	37.06	0.96	<LOD	282.4	25.3	<LOD	2689	6.36	2.08	1.53
	GrIS-16_17	H_{bio} ice	31.52	7.82	<LOQ	19.38	<LOD	0.374	118.86	1.32	<LOD	110.1	<LOD	<LOD	<LOD	5.47	2.64	1.77
	GrIS-16_28	H_{bio} ice	36.41	3.00	<LOQ	53.55	<LOD	<LOD	34.09	0.76	<LOD	<LOD	<LOD	<LOD	<LOD	5.76	2.04	1.50
	GrIS-16_13	DCC ice	33.65	5.06	<LOQ	24.37	<LOD	0.09	27.37	0.74	<LOD	<LOD	<LOD	<LOD	<LOD	6.09	1.71	1.36
	GrIS-16_18	DCC ice	18.84	4.51	<LOQ	20.64	<LOD	<LOQ	33.75	1.07	<LOD	1236	<LOD	<LOD	<LOD	5.50	2.82	1.89
	GrIS-16_29	DCC ice	49.30	5.69	18.06	63.50	<LOD	0.119	45.95	1.91	<LOD	<LOD	<LOD	<LOD	<LOD	5.08	3.73	2.30
	GrIS-16_12	Clean ice	37.21	0.97	<LOD	22.39	<LOD	<LOD	2.25	0.29	<LOD	<LOD	<LOD	<LOD	300	7.28	1.25	1.08
	GrIS-16_16	Clean ice	18.51	0.84	<LOD	<LOQ	<LOD	0.19	41.41	0.48	<LOD	<LOD	<LOD	<LOD	<LOD	5.99	1.52	1.25
	GrIS-16_27	Clean ice	20.40	3.04	<LOD	16.25	<LOD	<LOD	3.73	0.33	<LOD	<LOD	<LOD	108.2	<LOD	5.68	1.66	1.28
	GrIS-16_15	Supraglacial stream water	20.56	2.93	<LOD	<LOQ	<LOD	<LOD	<LOQ	0.32	<LOD	114.8	<LOD	<LOD	<LOD	6.38	1.78	1.45
	GrIS-16_23	Supraglacial stream water	23.73	3.20	<LOD	7.66	<LOD	<LOD	<LOD	0.22	<LOD	<LOD	<LOD	<LOD	<LOD	5.71	1.55	1.25
4b	GrIS-17-24	H_{bio} ice	20.61	7.02	<LOD	11.32	11.42	0.16	20.66	1.08	<LOD	<LOD	ND	ND	<LOD	6.79	2.48	1.75
	GrIS-17-26	H_{bio} ice	26.67	7.58	<LOD	13.56	11.03	0.18	16.60	1.38	<LOD	2953	ND	ND	<LOD	7.15	2.32	1.68

	GrIS-17-28	H_{bio} ice	29.21	9.92	<LOD	22.38	16.25	0.23	38.57	2.21	<LOD	289.6	ND	ND	<LOD	5.40	3.16	2.06
	GrIS-17-25	DCC ice	23.09	11.61	<LOQ	23.17	23.21	0.29	38.51	1.35	<LOD	10468	ND	ND	<LOD	5.20	2.15	1.64
	GrIS-17-33	Clean ice	46.72	<LOQ	<LOD	16.48	10.17	0.12	1.90	0.76	<LOD	4505	ND	ND	<LOD	5.26	3.46	2.19
5	GrIS-16_24(1)	H_{bio} ice	60.27	20.72	11.52	117.70	<LOD	0.37	84.94	2.02	<LOD	<LOD	<LOD	<LOD	<LOD	5.70	4.91	2.90
	GrIS-16_24(2)	H_{bio} ice	52.64	19.50	10.95	99.21	<LOD	0.33	83.85	2.43	<LOD	ND	ND	ND	ND	ND	ND	ND
	GrIS-16_25(1)	H_{bio} ice	79.39	4.91	12.14	45.74	<LOD	0.10	45.83	1.25	<LOD	<LOD	<LOD	<LOD	<LOD	5.70	3.24	2.09
	GrIS-16_25(2)	H_{bio} ice	53.33	4.59	12.14	40.71	<LOD	0.05	47.15	1.21	<LOD	ND	ND	ND	ND	ND	ND	ND
	GrIS-16_26(1)	H_{bio} ice	32.57	23.24	11.79	89.31	<LOD	0.32	71.35	1.82	<LOD	<LOD	<LOD	<LOD	<LOD	5.68	4.66	2.75
	GrIS-16_26(2)	H_{bio} ice	35.75	24.22	12.46	98.33	<LOD	0.33	74.33	1.92	<LOD	ND	ND	ND	ND	ND	ND	ND
		LOD/LOQ/Uncertainty	Na	Mg	Al	K	Ca	Mn	Fe	P	F	Cl	NO2	NO3	SO4			
	2016	LOD / µg・L^{-1}	4.26	0.171	1.46	4.67	3.81	0.015	0.242	0.37	<20	<100	<20	<100	<100			
		LOQ / µg・L^{-1}	14.2	0.569	4.88	15.6	12.7	0.051	0.808									
		% Uncertainty	0.874	0.876	1.57	1.07	1.56	0.448	0.686									
	2017	LOD / µg・L^{-1}	0.41	0.31	1.53	0.14	0.78	0.02	0.06	0.37	<20	<100	<20	<100	<100			
		LOQ / µg・L^{-1}	1.36	1.03	5.11	0.45	2.61	0.06	0.20									
		% Uncertainty	3.13	1.24	2.66	3.46	2.55	1.06	2.19									

LOD: level of detection, LOQ: level of quantification, ND: no data

Supplementary Table 9. Number of raw and processed sequences after each quality filtering step for 16S, ITS2 and 18S.

	16S						ITS2						18S					
Sample ID	Raw	Filtered	Denoised	Merged	Non-chimeric	Assigned to bacteria	Raw	Filtered	Denoised	Merged	Non-chimeric	Assigned to fungi	Raw	Filtered	Denoised	Merged	Non-chimeric	Assigned to algae
GrIS16-2	289712	190852	190852	164375	130211	83185	147601	52500	52500	50821	50788	49959	310981	252386	252386	248557	224527	125058
GrIS16-3	254898	170652	170652	129107	102516	80636	71630	20990	20990	18759	18759	17294	336077	272840	272840	268993	251714	166746
GrIS16-4	267420	183257	183257	156460	141775	17013	158228	58681	58681	52515	52155	49068	301594	246533	246533	243593	225056	148673
GrIS16-5	290907	192924	192924	170808	139361	91343	110908	41966	41966	38700	38211	37174	210774	175300	175300	173581	164441	106563
GrIS16-6	294136	201759	201759	186041	155293	80146	130211	43919	43919	38423	38297	37519	279352	229231	229231	227071	214042	145812
GrIS16-7	346321	223809	223809	198924	159024	118162	158973	63087	63087	59331	58238	55876	281200	231400	231400	229643	219069	154027
GrIS16-8	278728	183511	183511	161012	131176	87124	161976	66741	66741	59109	58480	55794	233059	191604	191604	189926	178340	118248
GrIS16-9	132033	84611	84611	82900	77400	1832	171365	57896	57896	51397	50552	50263	286241	230671	230671	228713	213856	170083
GrIS16-10	180400	124827	124827	98821	78626	67778	100239	31534	31534	31139	31139	30541	268286	210568	210568	207770	192551	125404

GrIS16-11	199531	135949	135949	132767	122045	5529	171851	67602	67602	60832	60210	51480	311224	246148	246148	241952	216760	135203
GrIS16-12	242133	159605	159605	141481	124580	42606	98455	28779	28779	28213	28174	28014	366600	292239	292239	289930	267170	175073
GrIS16-13	324772	209708	209708	190270	151530	114567	108322	32009	32009	29256	28183	27081	461687	370480	370480	366340	341858	218267
GrIS16-14	305051	194518	194518	158474	123925	119729	88071	26884	26884	25216	24597	23826	344456	263619	263619	260994	251561	89060
GrIS16-16	223194	149814	149814	136240	115550	47247	140556	46619	46619	40938	39678	38937	356179	286624	286624	284526	271745	234608
GrIS16-17	324911	216965	216965	192451	156402	96795	143461	50748	50748	44894	44113	41842	263309	210795	210795	208592	194510	127991
GrIS16-18	295450	189188	189188	167762	134466	103528	100178	32451	32451	28697	27834	27653	210713	169836	169836	166821	159286	94778
GrIS16-27	184582	124637	124637	118512	109465	14782	119924	44840	44840	43916	43101	42127	263969	214407	214407	213491	205146	180132
GrIS17-24	44426	33719	33719	29346	28684	806							92634	80112	80112	79444	76688	62883
GrIS17-25	17171	9855	9855	9689	9129	1232							68088	58606	58606	58309	56268	47567
GrIS17-26	110135	82856	82856	82266	80477	1409							70931	61781	61781	61376	59323	50515
GrIS17-27	67170	48761	48761	43307	36383	36262							87598	73744	73744	73112	71848	7161
GrIS17-28	131297	99794	99794	97472	92206	15636							76839	66105	66105	65430	63044	51840
GrIS17-29	114759	87529	87529	86377	83186	5801							132476	113532	113532	112795	108243	89390
GrIS17-33	58038	43210	43210	42345	39479	9248							106162	93596	93596	93421	93410	90165
GrIS17-34	85071	66375	66375	65878	63054	7142							142082	124158	124158	123049	118368	98835
GrIS17-35	64118	48428	48428	47513	45430	7652							164414	144185	144185	142948	136829	108919

Supplementary Table 10. Table shows the full bacterial community composition with the taxonomic assignments of each ASV on the lowest possible level. Values represent the relative abundances of the 16S ASVs in percentage of the total number of sequences and collapsed on the species level. Values are rounded to one decimal place, thus “<” represents relative abundance values < 0.05 and > 0.

			Year	2016															2017					
			**Site**	**2**	**3**	**3**	**3**	**4a**	**4a**	**4a**	**4a**	**4a**	**4a**	**4a**	**4a**	**4a**	**4a**	**4a**	**4b**	**4b**	**4b**	**4b**	**4b**	**4b**
			**Sample ID**	**16-2**	**16-3**	**16-4**	**16-10**	**16-5**	**16-6**	**16-7**	**16-8**	**16-12**	**16-13**	**16-14**	**16-16**	**16-17**	**16-18**	**16-27**	**17-27**	**17-28**	**17-29**	**17-33**	**17-34**	**17-35**
Phy-lum	**Class**	**Order**	**Family, Genus, Species**																					
**Acidobacteria**	Acido-bacteriia	Acidobacteriales	Acidobacteriaceae	22.5	15.8	19.9	18.5	3.4	3.1	2.7	3.3	28.0	4.8	5.3	3.8	4.4	2.1	4.0	0.4	4.9	3.1	1.5	5.3	3.9
			Acidobacteriaceae	0.5	0.4	0	0.4	0.1	0.1	<	0.1	<	<	0.1	0.1	0.1	<	0	0.1	0	0	0	0	0
	Solibacteres	Solibacteriales		0	0.1	0	0.1	0	0	<	0	0	0	0	0	0	0	0	0	0	0	0	0	0
				0	0	0	0	0	<	0	0	0	0	<	0	<	0	0	0.1	0	0	0	0	0
			Solibacteraceae	0	0.7	0	0.8	<	<	0	<	0	0	<	0	0	0	0	<	0	0	0	0	0
			Solibacteraceae; Candidatus Solibacter	0	1.3	0	0.9	0.1	0.2	0.1	0.3	0.5	0.1	0.4	0.6	0.2	0.2	0.3	0.3	0.3	0.3	0.1	0	0.1
**Actinobacteria**	Acidimicrobiia	Acidimicrobiales		0	1.1	0	0.6	0.1	0.2	0.4	0.2	<	0.2	0.3	0.6	0.1	0.4	0.1	0.7	0.1	0	0.8	0	0.3
			EB1017	0	0	0	0	0	0	0	0	0	0	0	0	0	0	0	0	0	0	0.2	0	0
	Actinobact[illegible]			0	0.1	0	0.1	0	<	<	0.1	0	0	0	<	<	<	0	0.1	0	0	0	0	0

		Actinomycetales		0	0	0	0	0	0	0	0	0	0	0	0	0	0	0	0	0	0	0	0	0
			ACK-M1	1.6	2.8	0	3.0	0.3	0.5	0.1	0.9	0.2	0.3	1.2	0.7	0.4	0.3	0.3	0.8	0.3	0	0.4	0.1	0.4
			Corynebacteriaceae; Corynebacterium	0	0	0	0	0	0	0	0	0	0	0	0	0	0	0	0	0	0	0.9	0	0
			Intrasporangiaceae	0	0	0	0	<	<	<	<	0	0	<	0.1	<	0	0	0	0	0	0	0	0
			Intrasporangiaceae	0	0	0	0	0	0	0	0	0	0	0	0	0	0	0	0	0	0	0	0	0
			Microbacteriaceae	3.9	0.4	1.0	0.6	<	0.1	<	0.1	0	0.1	0.1	0.1	<	<	0	0.3	0.5	0	0.2	0	0.2
			Microbacteriaceae; Salinibacterium	14.8	8.1	0.5	8.1	0.4	0.7	0.4	0.5	3.3	1.0	0.5	1.1	0.8	0.7	1.7	0.4	1.8	0.7	3.1	1.5	2.7
			Microbacteriaceae; Salinibacterium	0.5	0.1	0	0.3	0	0	0	0	0	0	0	0	0	0	0	0.1	0	0	0	0	0
			Nakamurellaceae	0	0.2	0	0.2	0	0.1	<	<	0	<	<	0	0	0	0	0	0	0	0	0	0
			Propionibacteriaceae; Propionibacterium; P. acnes	0	0	0	0	0	0	0	0	0	0	0	0	0	0	0	0	0	0	0.4	0	0
			Sporichthyaceae	0	0.8	0.1	1.3	0.1	0.1	0.1	0.1	0.1	0.1	<	0.3	0.2	0.2	0.3	0.2	0.2	0.2	0.8	0	0.5
**Armatimonadetes**	Armati-monadia	Armatimonadales	Armatimonadaceae	0	0.5	<	0.5	0.7	0.6	0.4	0.6	0.1	0.5	0.6	0.2	0.2	<	0.1	0.1	0.2	0.5	0	0	0.2
		FW68		11.0	21.8	8.6	22.7	10.8	9.5	7.5	10.6	13.3	12.1	10.1	8.4	13.3	9.9	6.1	1.0	29.5	40.8	2.8	46.5	14.7
	Chthono-monadetes	Chthono-monadales	Chthonomonadaceae	0.2	0.3	0	1.1	0.4	0.3	0.6	0.8	0.4	0.4	0.5	0.5	0.3	0.3	0.2	0.4	<	0.3	0.1	0	0.2
**Bacteriodetes**	Bacte-roidia			0	<	0	0	0.1	<	0.1	<	0.1	0	<	0.1	<	0	0.3	0.6	0	0	0	0	0
				0.1	<	0.9	0	0.1	<	0	<	0.2	0	0	0.2	<	0	0.3	0	0	0	0	0	0
	Cytophagia	Cytophagales	Cytophagaceae; Cytophaga	0	0	0	0	0	0	0	0	0	0	0	0	0	0	0	0.3	0	0	0	0	0
			Cytophagaceae; Flectobacillus	0	0	0	0	0	0	0	0	0	<	0	0	<	<	0	0	0	0	0	0	0
			Cytophagaceae; Hymenobacter	<	0	2.1	<	<	0	<	0	0.2	<	0	0.2	0.1	<	0.4	0	0	0	0	0	0
			Chitinophagaceae; Flavisolibacter	0	0	0	0	0	0	0	0	0	0	0	0	0	0	0	0	0	0	0	0	0
**Chloroflexi**	Anaero-lineae			0	0	0	0	0.6	0.7	0.7	0.9	<	0.4	0.4	1.0	0.6	0.8	0.6	0.4	0.2	0.1	0.6	0.2	0.3
				0	0	0	0	0.8	0.5	1.1	0.8	0	0.4	1.0	0.5	0.5	0.6	0.1	0.9	<	0.1	0.2	0	0.1
		SBR1031	oc28	0	0	0	0	0.5	0.5	0.8	0.9	0.1	0.5	0.7	0.7	0.5	0.9	0.3	1.9	0.1	0.2	0.4	0	0
	Ktedono-bacteria	Thermogemma-tisporales	Thermogemmatisporaceae	<	<	0	0	25.2	28.6	28.2	23.4	8.1	23.5	22.7	28.6	23.6	28.9	23.0	32.6	17.7	6.4	16.9	5.9	15.7
	Thermo-microbia	Ellin6537		0	0	0	0	0.1	0.1	0.1	0.1	0	0	<	0.2	0	0.1	<	0.1	0	0	0	0	0
**Cyano-bacteria**	Synecho-Cocco-phycideae			0	0.2	0.6	0.1	15.5	13.8	16.4	12.0	8.9	19.3	12.7	15.4	14.1	12.0	23.4	16.7	4.4	5.5	26.6	9.2	12.8
		Pseudanabaenales	Pseudanabaenaceae; Leptolyngbya	0	<	0	0.6	7.7	6.9	9.4	9.0	0.5	6.3	9.1	7.1	4.4	10	2.3	15.6	1.4	1.3	2.7	0.3	1.7
		Synechococcales	Chamaesiphonaceae	0	0.3	0	2.3	2.1	2.0	3.2	2.5	0	1.2	1.2	0.4	1.1	1.8	0.1	2.5	0.7	0.9	2.3	0.1	0.9
**Firmi-cutes**	Clostri-dia	Clostridiales	Clostridiaceae; Clostridium; C. bowmanii	0.1	0.3	0	0.1	0	0	0	0	0	0	0	0	0	0	0	0	0	0	0	0	0
**Gemma-timonadetes**	Gemm-1			0	0	0	0	0	0	0	0	0	0	0	0	0	0	0	0	0	0	0.4	0	0
	Gemma-timona-	N1423WL		0	0	0	0	0	0.1	<	0.2	0	0	0.1	0	0	<	0	<	0	0	0	0	0

**OD1**				0	0.1	0.1	0.1	0	<	<	<	0	<	0	0	<	0	0	0	0.1	0.1	0	0	0
**Plancto-mycetes**	Plancto-mycetia	Gemmatales		0	0	0	0	0.1	0.1	0.3	0.3	0	0.1	0.1	0	0.1	0.1	0	0.5	0	0	0	0	0
			Gemmataceae; Gemmata	0	0	0	0	0.1	0	0	0.1	0	0	0	<	0.1	0	0	0.1	0	0	0	0	0
			Isosphaeraceae	0	0.5	0.1	0.8	0.3	0.2	0.1	0.2	0.1	0.1	<	0.3	0.3	0.3	0.1	<	0.1	0	0	0	0.3
**Proteobacteria**	Alphaproteobacteria	Caulobacterales	Caulobacteraceae	0	<	0	0.2	<	0.1	0.1	0.1	<	0	0.1	0.1	0.1	0.1	0.1	0	0	0	0	0	0
			Caulobacteraceae; Phenylobacterium	0	0.6	0	0.2	0	0	0	0	0	0	0	0	0	0	0	0	0	0	0	0	0
		Rhizobiales		0	0.2	0	0.3	0	0	0	0	0	0	0	0	0	0	0	0	0	0	0	0	0
			Bradyrhizobiaceae	0	<	0	0.1	0.2	0.3	0.3	0.2	<	0.2	0.2	0.6	0.2	0.3	0.2	0.5	0.2	0.2	0	0	0.2
			Bradyrhizobiaceae; Bosea; B. genosp	0.1	0.1	0	0.3	0	0	0	0	0	0	0	0	0	0	0	0	0	0	0	0	0
			Methylocystaceae	0	0	0	0	0.3	0.2	0.1	0.2	0	0	0.1	0.1	0.1	0	0.1	0.5	0	0.4	0	0	0
			Rhizobiaceae; Agrobacterium	0	0	0	0	0	0	0	0	0	0	0	0	0	0	0	0	0	0	0	0	0
		Rhodospirillales	Acetobacteraceae	25.8	19.0	61.0	17.3	23.2	22.2	21.1	18.1	25.0	23.3	14.9	12.0	24.5	23.1	24.2	15.6	32.4	30.2	33.3	28.7	38.4
		Ricksettsiales		0	0	0	0	0	0	0	0	0	0	0	0	0	0	0	0	<	0	0	0.1	0.1
		Sphingomonadales		6.6	6.8	3.4	3.1	1.0	0.7	0.6	1.4	1.7	0.8	1.5	1.9	1.1	0.7	1.9	0.2	1.8	5.4	0	1.2	1.3
				0.1	1.2	0.3	1.3	2.1	3.0	1.5	4.8	1.2	1.0	6.7	5.8	3.3	1.5	2.8	1.4	0.8	1.2	1.8	0.2	1.4
			Sphingomonadaceae; Novosphingobium	2.3	0.5	0	0.4	0	0	0	0	0	0	0	0	0	0	0	0.3	0	0	0	0	0
			Sphingomonadaceae; Novosphingobium	0	0	0	0	0	0	0	0	0	0	0	0	0	0	0	0	0	0	0	0	0
			Sphingomonadaceae; Sphingomonas; S. echinoides	1.0	0	1.1	0	0	0	0	0	0	0	0	0	0	0	0	0	0	0	0	0	0
			Sphingomonadaceae; Zymomonas	0	0.4	0.2	0.5	0.7	0.7	0.3	0.5	0.2	0.5	0.7	0.1	0.1	0	0.1	0.3	0.3	0.6	0	0	0.2
			Comamonadaceae; Rhodoferax	0	0	0	0	0	0	0	0	0	0	0	0	0	0	0	0	0	0	0	0	0
	Delta-Proteo-bacteria	Bdellovibrionales	Bdellovibrionaceae; Bdellovibrio	0.4	0.7	0	0.4	0.1	0.3	0.2	0.4	0	0.2	0.4	<	<	<	<	0.1	0	0	0	0	0
**TM7**	SC3			0	0.3	0	0.2	0	0	0	0	0	0	0	0	0	0	0	0	0	0	0	0	0
	TM7-1			0	0	0	0	0	<	0	<	0	0	0.1	0	<	<	0	<	0	0	0	0	0
	TMT-3	EW055		8.1	1.2	0	0.8	0	0	0	0	<	0	0	0	0	<	0	0	0	0	0	0	0
**Verruco-microbia**	[Sparto-bacteria]	[Chthonio-bacterales]	[Chthoniobacteraceae]; Candidatus Xiphinematobacter	0	0	0	0	0	0	0	0	0	0	0	0	0	0	0	0.3	0	0.1	0	0	0
**WPS-2**				0.4	13.0	0	11.5	2.5	3.4	2.5	6.0	7.9	2.4	7.8	8.4	4.7	4.5	6.8	3.1	1.8	1.7	3.6	0.9	3.4
**WS6**	B142			0	0	0	0	0.2	<	0.2	<	0	0.1	0.2	0.1	0.1	<	0	0.1	0	0	0	0	0

Supplementary Table 11. Table shows the full eukaryotic community composition collapsed into higher eukaryotic taxonomic groups. Values represent the relative abundance of the 18S ASVs in percentage of the total number of sequences and collapsed on the species level. Values are rounded to one decimal place, thus “<” represents relative abundance < 0.05 and > 0.

		Year	2016																	2017								
		**Site**	**2**	**2**	**3**	**3**	**3**	**3**	**4a**	**4a**	**4a**	**4a**	**4a**	**4a**	**4a**	**4a**	**4a**	**4a**	**4a**	**4b**	**4b**	**4b**	**4b**	**4b**	**4b**	**4b**	**4b**	**4b**
		**Sample ID**	**16-2**	**16-9**	**16-3**	**16-4**	**16-10**	**16-11**	**16-5**	**16-6**	**16-7**	**16-8**	**16-12**	**16-13**	**16-14**	**16-16**	**16-17**	**16-18**	**16-27**	**17-24**	**17-25**	**17-26**	**17-27**	**17-28**	**17-29**	**17-33**	**17-34**	**17-35**
D0	**D1**	**D2**																										
**Amoebozoa**	Discosea	Flabellinia	0	0	0	0	0	0	0	0	0	0	0	0	0	0	0	0	0	0	0	0	0.1	0	0	0	0	0
	Gracili-podida	Flamella	0	0	0	0	0	0	<	<	<	<	0	<	0.1	0	0	<	0	0	0	0	0.1	0	0	0	0	0
	Tubulinea	Euamoebida	0	0	2.8	0	0.3	0	0	0	0	<	<	0	0.2	0	<	0	0	0	0	0	0.2	0	0	0	0	0
			0	0	0	0	0	0	0.2	0.5	0.4	<	0	<	0.6	<	<	<	0	0	0	0	9.2	0	0	<	0	0
**Archae-plastida**	Chloro-plastida	Charophyta	55.6	80.4	58.7	65.1	51.6	53.9	61.2	65.0	67.7	62.1	65.4	57.3	25.8	85.5	64.5	39.7	87.5	79.9	84.4	84.5	1.9	78.6	80.7	96.1	80.5	78.3
		Chlorophyta	0.2	0.3	7.9	1.6	14.0	8.4	3.8	3.1	2.9	4.3	0.3	6.5	9.7	1.1	2.3	17.5	<	2.0	0.2	0.7	7.5	3.6	1.7	0.3	3.0	1.5
**Opisthokonta**	Holozoa	Metazoa	<	<	<	<	<	<	10.9	7.4	6.6	10	<	1.2	23.9	<	4.4	14.9	0	0	0.1	<	56.6	<	<	0.1	<	0
	Nuclet-myceae	Fungi	34.1	14.8	16.9	27.1	26.1	23.1	19.2	20.8	19.7	20.5	34.1	30.4	32.3	10.7	19.2	14.9	11.4	16.0	15.2	13.8	17.6	13.6	16.0	2.3	11.5	19.6
		Nucleariidae and Fonticula group	<	0	0	0	0	0	0	0	0	0	0	0	<	0	0	0	0	0	0	0	<	0	0	0	0	0
			0	0	0	0	0	0	0	0	0	0	0	0	0	0	0	0.1	0	0	0	0	0.1	0	0	0	0	0
**SAR**	Alveolata	Ciliophora	1.0	0.4	1.8	3.1	0.4	2.4	0.6	1.0	0.8	1.2	0.1	1.0	3.9	2.2	8.4	5.3	0.2	0.4	0	0.1	1.8	0.6	<	0.2	0.5	0.1
		Dinoflagellata	2.6	1.5	7.4	0.1	0.4	0.3	3.4	1.4	1.2	1.1	0.1	1.4	0.6	0.2	0.4	0.1	0	1.6	0.1	0.6	1.7	3.2	1.0	0.7	4.2	0.6
	Rhizaria	Cercozoa	6.2	2.5	4.0	3.0	7.0	11.9	0.6	0.7	0.6	0.6	<	1.8	2.3	0.2	0.7	4.9	0.4	0.1	<	0.2	2.3	0.4	0.4	0.1	0.2	<
	Stramenopiles	Bicosoecida	0	0	<	0	<	0	0	0	0	0	0	0	<	0	0	0	0	0	0	0	0.1	0	0	0	0	0
		Labyrinthulo-mycetes	0	0	0	0	0	0	0	0	0	0	0	0	0	0	0	0	0	0	0	0	0.1	0	0	0	0	0
		Ochrophyta	0.1	<	0.2	<	0.2	<	0.1	0.1	0.1	0.2	<	0.4	0.6	<	0.1	2.7	0.5	<	<	0.1	0.7	0.1	0.2	0.2	0.1	<
		Peronosporo-mycetes	0	0	0.3	0	<	0	<	<	<	<	0	0	0.1	<	0	0	0	0	0	0	0.1	0	0	0	0	0

Supplementary Table 12. Table shows the fungal community composition with the taxonomic assignments of the ten most abundant ASV on the lowest possible level. The representative sequences were blasted against NCBI and the closest accession number with the respective similarity were recorded. If several hits shared the similarity one hit was chosen as an example (“e.g.”). Values represent the relative abundance of the ITS2 ASVs in percentage of the total number of sequences. Values are rounded to one decimal place, thus “<” represents relative abundance values < 0.05 and > 0.

		Year	2016																
		**Site**	**2**	**2**	**3**	**3**	**3**	**3**	**4a**	**4a**	**4a**	**4a**	**4a**	**4a**	**4a**	**4a**	**4a**	**4a**	**4a**
ASV ID	**Taxonomic assignment**	**Accession number (similarity)**	**16-2**	**16-9**	**16-3**	**16-4**	**16-10**	**16-11**	**16-5**	**16-6**	**16-7**	**16-8**	**16-12**	**16-13**	**16-14**	**16-16**	**16-17**	**16-18**	**16-27**
**07892df564bfb39552b9a0e574361226**	Basidiomycota sp.	MK045389.1 (91.36%)	49.7	42.1	0	69.7	30.1	70.7	48.6	49.8	45.4	38.0	52.5	30.4	17.3	33.0	32.1	20.5	75.6
**dd187f27730de8ee9c227cba215cb89a**	Microbotryomycetes sp. strain V155	MF615028.1 (91.80%)	0.3	1.4	0	1.0	0.9	4.5	6.8	12.7	4.9	11.9	1.9	9.1	7.1	12.1	11.1	12.5	0.7
**0862d42a8aa9048d06af0daa70c29b5a**	No blast hit (1)		0	0	0	0	<	0	9.6	5.7	12.2	0	0.5	13.0	31.4	5.2	3.9	5.0	0.2
**eb54f47b0264c9e00e133ae90bc541c6**	No blast hit (2)		2.5	4.6	1.1	10.9	1.2	10.6	1.8	2.0	2.0	4.9	0.3	1.7	1.7	8.2	9.1	6.6	0.1
**1316f0da74fff2d47e02f71f6485dc52**	Pezizomycotina sp./Helotiales sp. (1)	e.g., MF615085.1/ MF043974.1 (100%)	17.0	6.2	9.9	0.2	12.4	0.2	1.5	0.8	1.5	0.9	3.1	2.2	2.3	1.7	1.8	4.3	0.2
**e4d8c7cc2cd7be1e50beb02b1523e504**	Pezizomycotina sp./Helotiales sp. (2)	e.g., MF615085.1/ MF043974.1 (100%)	13.9	7.9	8.2	0.3	15.8	0.1	1.3	0.8	1.6	1.1	2.5	1.9	2.6	1.6	1.6	3.0	0
**b95d68ad46eef17c47774f6c74ab71bd**	Rhodotorula sp.	e.g., MK671639.1 (100%)	0	0	0	0	0	0	2.7	2.4	2.8	2.9	1.2	8.9	13.1	1.0	6.1	17.3	0.1
**84ff4cf007fda6b6514d411727fd0c44**	Herpotrichiellaceae sp.	KF636410.1 (97.30%)	0	0	0	0	0	0	3.6	2.6	4.9	3.3	5.1	10	7.2	9.0	4.0	8.1	0.4
**c0f393e0d357cc4f61bf1a814acb82f4**			0	0	0	0	0	0	10.9	10	10.1	8.6	4.0	3.5	2.3	7.3	6.9	7.0	0.9
**d9250a2f909c0c94e6ebe43baa84f521**			1.4	1.4	0.8	2.3	0.9	1.9	4.2	0	4.1	3.5	8.3	4.7	2.2	4.0	4.2	2.3	2.4
**549304d1b3e26af65faf1af31d0d48c6**			0	0	0	0	0	0	3.6	1.8	4.1	3.4	0.3	6.7	4.7	2.2	0.6	0.7	0.3
**d4fcebaee77b8358282fc7e2f9a99eb4**			0	0	28.4	0	0	0	0	0	0	0	0	0	0	0	0	0	0
**f261fc44c08d54e6bcd29961c6c37c45**			3.4	1.9	0	0	0	0	0.4	1.9	0.7	0.8	0	0.3	0.4	2.1	7.5	3.8	0
**cab5746aafd00c2d8572c0958e3c9638**			0	<	0.1	0.4	0.1	2.0	0.7	0.5	0.5	0.6	1.6	0.7	0.8	0	0.9	1.2	12.1
**e9cda2a8a48107d128e3fb4bf1c22a35**			0	0	0	0	0	0	1.4	1.1	1.7	2.2	0.5	2.6	3.4	1.2	1.6	5.0	0.2
**91b33e406a755eede56fd9f97651ef9e**			0	0	19.3	0	0	0	0	0	0	0	0	0	0	0	0	0	0
**c5ceaed3c7dc25fc7ed25c55183f11f5**			4.2	9.5	0	0.2	0	0	0	0	0	0	0	0	0	0	0	0	0
**3e89eec9ab87565f807d72ee27715518**			0	0	0	0	0	0	0	0	0	12.2	0	0	0	0	0	0	0
**6b72280905b643c0fb5974d2d77879c8**			0	0	8.0	0	2.4	0	0	0	0	0.1	0	0	0	0	0	0	0
**9d4ff72903eb01cdc573aaf09e8394c9**			0	0	2.3	0	5.9	0	<	<	0.1	<	0.1	0.3	0.2	0.1	0.1	0.5	0
**95e441833de252ad845c1f5752375d9d**			0	0	0	6.3	0	3.1	0	0	0	0	0	0	0	0	0	0	0
**0d2969f56d97cef21d15f56f8bd3dd51**			0	0	9.3	0	0	0	0	0	0	0	0	0	0	0	0	0	0
**f5cfe14fb1a601ef5615d0c3715ec745**			0	0	0	0	9.0	0	0	0	0	0	0	0	0	0	0	0	0
**f8a57d6dfda711ad1a5786f3c2abda94**			0.2	0.1	0	0	<	<	0.1	0	0.1	0.1	5.5	0	0	0.5	0.1	0.2	1.9
**18c811a9d8b5e888a44d72109e188857**			0	0	0	0	0	0	0.1	2.1	0.4	2.3	0	1.6	0.8	<	0.9	0	0
**8c2311ec87b85766c9910af02e43c178**			0	0	0.6	0	6.6	<	0	0	0	0	0.1	0	0	0	0	<	0
**75e2fa7712d9c9c81e157e7c32d504cc**			0	0	0	0	6.2	0	0	0	0	0	0	0	0	0	0	0	0
**900939e27331f239c40d3a102f6f32f9**			0	0	0	0	0	0	0	0	0.1	<	4.6	0	0	0.2	<	0	0.1
**896af8512258ac794dc03277bcc5bc76**			1.1	0.5	1.1	0.3	1.2	0	0	0	0	0	0.4	0.1	0	0	0.1	0	0
**7b79301ea0e9678345165c745c76fe59**			0	0	0	0	0	0	0	0.1	0	0	3.3	0	0	0.2	0	0	0.9
**ea86391d31c8c4ca5059c7d399c53c47**			0	0	0	0	0	0	0	4.5	0	0	0	0	0	0	0	0	0
**f55a499dd3bf1b72aa5ca1f00e446a9a**			0.1	<	<	0.7	0	0.2	0	0.1	0.2	0.2	2.0	0.2	0	0.4	0.3	0.1	0
**e6e295487c275c90bb0b57b1f3748444**			0.7	0.3	0.9	0.2	1.3	0.2	<	0	0	0	0.3	0	0	0.1	0	0.1	0
**b2d815f764528856c81093c0e83860e3**			0	0	0	0	0	0	0.4	0.1	1.0	0.3	0	0	0	0.2	2.1	0	0
**506520fbaa88ae977b968a7123313ae5**			0	0	0	0	0	0	0.1	0.1	0.1	0.1	0	0.2	0.1	0.5	2.5	0.1	0
**1cd2c551fd1296a9ef67e047ee74c183**			0	0	3.2	0	<	0	<	0	0	0	0	0	<	0	0	0	0
**49da64cfc7c09efb3711c8c38d1e9614**			0.5	2.1	0	0.3	0	0.2	0	0	0	0	0	0	0	0	0	0	0
**e27312f054bc2f7af83b15263bff1ced**			0.4	2.1	0	0.3	0	0.2	0	0	0	0	0	0	0	0	0	0	0
**117de4dac3c76149f0cce26098af7680**			0	0.1	<	0.8	0	2.1	0	0	0	0	0	0	0	0	0	0	0

**901b96de056ba115dd4bb2f48f135ede**	0	0	0	0	0.1	0	0.2	0.2	0.2	0.3	0	0.3	0.6	0.2	0.3	0.7	0
**a352b73df462a7cd1d641eadc54c803a**	0.5	2.3	0	0.1	<	0	0	0	0	0	0	0	0	0	0	0	0
**7528e837bba42e5d7e6dbefa03f32cd6**	0	2.9	0	0	0	0	0	0	0	0	0	0	0	0	0	0	0
**13a3e4c112e47f0ba6a4a7d9a4fea0e8**	0.3	1.2	0.1	0.2	0	0.5	0	0	<	<	0.3	0.1	0	0	0.1	0	<
**16588c840792e0c4d1ed78bf8066e621**	0	0	0	0	0	0	0	0	0	0	0	0	0	1.6	0	0	1.1
**2ca1fdf180e78c761f651c5fccca72df**	0.6	2.0	0	0.1	0.1	0	0	0	0	0	0	0	0	0	0	0	0
**58618f5d53426428f663886e4f4a4d7d**	0	0	0	2.5	0.1	0	0	0	0	0	0	0	0	0	0	0	0
**46245d37bc4b998666f3d9800f9862fe**	0.2	2.3	0	0	0	0	0	0	0	0	0	0	0	0	0	0	0
**d2dfc392d3995163972b46f5d6be640e**	0.6	1.8	0	0	0	0	0	0	0	0	0	0	0	0	0	0	0
**382e5d645369e294afa4f98c9094ee39**	0	0.5	0	0.8	0	0.6	<	<	<	<	0	0.2	0.1	0	0.1	0	0
**616115979f7af934de68e045e5dfbcbb**	0	0	0	0	0	0	0	0	0	0	0	0	0	2.2	0	0	0
**Other**	2.6	6.6	6.5	2.4	5.7	2.9	1.5	0.6	1.1	2.3	1.5	1.5	2.0	5.2	1.7	1.0	2.6

Supplementary Table 13. Table shows the full algal community composition with the taxonomic assignments of each ASV on the lowest possible level. Values represent the relative abundance of the 18S ASVs in percentage of the total number of sequences. All ASVs were blasted against NCBI and the closest accession number with the respective similarity were recorded. If several hits shared the similarity one hit was chosen as an example (“e.g.”). Values are rounded to one decimal place, hence “<” represents relative abundance < 0.05 and > 0.

	Year	2016																	2017							
	**Site**	**2**	**2**	**3**	**3**	**3**	**3**	**4a**	**4a**	**4a**	**4a**	**4a**	**4a**	**4a**	**4a**	**4a**	**4a**	**4a**	**4b**	**4b**	**4b**	**4b**	**4b**	**4b**	**4b**	**4b**
	**Sample ID**	**16-2**	**16-9**	**16-3**	**16-4**	**16-10**	**16-11**	**16-5**	**16-6**	**16-7**	**16-8**	**16-12**	**16-13**	**16-14**	**16-16**	**16-17**	**16-18**	**16-27**	**17-24**	**17-25**	**17-26**	**17-28**	**17-29**	**17-3**	**17-34**	**17-35**
Taxonomic assignment	Accession number (similarity)																									
**Ancylonema nordenskioeldii**	AF514397.2 (100%)	31.5	8.7	6.7	12.1	12.5	12.2	59.9	69.4	70.4	75.5	51.1	31.0	37.3	91.2	37.5	46.9	93.0	65.3	82.9	55.0	55.5	84.1	96.5	60.6	47.4
**Ancylonema nordenskioeldii**	AF514397.2 (98.8%)	0	0	0.1	<	<	<	<	<	0.1	<	0.2	<	<	<	0.1	<	<	0	<	0	0	<	0	0	<
**Ancylonema nordenskioeldii***	AF514397.2 (99.6%)	66.7	89.4	79.6	84.4	65.0	73.6	32.6	24.4	24.1	16.3	47.5	57.3	32.4	6.4	58.2	18.6	5.8	30.5	16.0	42.2	38.8	12.5	2.2	34.3	48.8
**Ancylonema nordenskioeldii**	AF514397.2 (100%)	0	0	0	0	0	0	0	0	0.1	0.1	0.1	0	0	0.2	0	0	0.2	0.2	0.2	0	0	0.3	0	0.2	0.1
**Ancylonema nordenskioeldii**	AF514397.2 (99.1%)	0	0	0.2	0.1	0.3	0.1	0	0	0	0	0	0.2	0	0	0	0	0	0	0	0.2	0.1	0	0	0.1	0.2
**Ancylonema nordenskioeldii**	AF514397.2 (99.7%)	0	0	0	0	0	0	0.5	0.6	0.6	0.5	0	0.2	0.3	0.1	0.3	0.5	0	0.4	0.2	0.3	0.3	0.4	0	0.3	0.4
**Ancylonema nordenskioeldii**	AF514397.2 (98.1%)	0	0	0.1	<	0.1	<	0	0	0	0	0	0.1	<	0	<	0	0	<	0	0.2	0.1	0	0	0.1	0.1
**Ancylonema nordenskioeldii**	AF514397.2 (99.1%)	0	0	<	<	<	<	<	0.1	<	0.1	0.2	<	<	<	0.1	<	<	0	0	0	0	<	<	0	<
**Ancylonema nordenskioeldii**	AF514397.2 (99.4%)	0	0	0	0	0.4	0	0	0	0	0	0	0	0	0	0	0	0	0.1	0	0.4	0.3	0	0	0.3	0.3
**Ancylonema nordenskioeldii**	AF514397.2 (99.1%)	0.1	0.2	0.1	0.2	0	0.1	0	0	0	0	0	0	0	0	0	0	0	0	0	0	0	0	0	0	0
**Ancylonema nordenskioeldii**	AF514397.2 (99,7%)	0	0	0	0	0	0	0.1	0.1	0.1	0.1	0.2	0	0	0.6	0	0	0.2	0.1	0.2	0	0	0.2	0.4	0.1	0.1
**Ancylonema nordenskioeldii**	AF514397.2 (99.4%)	0.4	0.4	0.3	0.4	0.3	0.4	0	0	0	0	0.1	0.2	0	0	0.2	0	0	0.1	0	0	0	0	0	0.1	0.1
**Ancylonema nordenskioeldii**	AF514397.2 (99.7%)	0.1	<	<	0	0	0	0.1	0.1	0.1	0	<	0.1	0	0.2	<	0	0.2	0.1	0	0	0	0.1	0.2	0.1	0.1
**Ancylonema nordenskioeldii**	AF514397.2 (99.4%)	0.1	0.2	0.1	0	0	0	0.1	<	<	0	0.1	0.1	0	<	0.1	0	<	0.1	0	0	0	<	<	0.1	0.1
**Ancylonema nordenskioeldii**	AF514397.2 (99.4%)	0.5	0.6	0	0	0	0	0	0	0	0	0	0	0	0	0	0	0	0	0	0	0	0	0	0	0
**Ancylonema nordenskioeldii**	AF514397.2 (98.5%)	0	0	0.3	0.2	0.3	0.2	0	0	0	0	0	0.2	0.1	0	<	0	0	0.1	0	0.2	0.1	0	0	0.1	0.1
**Ancylonema nordenskioeldii**	AJ579339.1 (99.1%)	0	0	0	0	0	0	0.3	0.3	0.3	0.3	0	0	0.2	0	0	0.3	0	0.4	0	0.3	0.2	0.2	0	0	0.3
**Botrydiopsis constricta**	AJ579339.1 (100%)	0	0	0	0	0	0	0	0	0	0	0	0	0	0	0	3.7	0	0	0	0	0	0	0	0	0
**Chlainomonas sp.**	e.g., MF803745.1 (99.1%)	0.1	0.2	0.1	0.6	0.2	1.5	2.2	2.0	1.5	3.4	0.1	2.3	0.6	0.5	2.1	0.5	<	2.3	0.2	0.7	3.5	1.2	0	3.3	1.6
**Chloroidum sp.**	MH807079.1 (100%)	0	0	0	0	0	0	0	0	0	0	0	0	0	0	0	1.8	0	0	0	0	0	0	0	0	0
**Chloromonas spp.**	Several (100%)	0	0	0	0.1	0	0.5	0	0	0	0	0	0	0	0	0	4.9	0	0	0	0	<	0	0	<	0
**Chloromonas spp.**	Several (100%)	0	<	<	<	0	0.2	0	0	0	0	0	0	0	0	0	1.7	0	0	0	0	0	0	0	<	0
**Chloromonas spp.**	e.g., AB906350.1 (99.4%)	0	0	0	0	0	0	0	0	0	0	0	0	0	0	0	1.3	0	0	0	0	0	0	0	0	0

**Chloromonas spp.**	e.g., LC012710.1 (100%)	0	0	0	0	0	0	0	0	0	0	0	<	<	<	<	12.8	0	0	0	0	0	0	0	0	0
**Hydrurus sp.**	HE820740.1 (100%)	0	0	0	0	0	0	0	0	0	0	0	0.4	0	0	<	<	0	0	0	0	0	0	0.1	0	0
**Hydrurus sp.**	HE820740.1 (100%)	0	0	0	0	0	0	0	0	0	0	0	0.1	<	0	<	0	0	<	0	0	<	<	0.1	0	0
**Ochromonas sp.**	MG674914.1 (100%)	<	0	0.2	<	0	<	0	0.1	<	0.4	<	<	1.1	0	0	0	0.1	0	0	<	<	0	0	0	0
**Ochromonas sp.**	MG674914.1 (99.7%)	0	0	0	0	0	0	0.1	<	0.1	0	0	0	0.1	0	0.1	<	0.4	0	<	0	0	0.2	0	0	0
**Ploeotila sp. CCCryo086-99**	GU117586.1 (98.5%)	0	0	0.5	0	0.2	0	0	0	0	0	0	0	0.1	0	0	0	0	0	0	0	0	0	0	0	0
**Ploeotila sp. CCCryo086-99**	GU117586.1 (99.1%)	0	0	0.2	0	<	0	<	0.1	<	<	0	<	0.7	0	0	0	0	0	0	0	0	0	0	0	0
**Raphidonema sp./Koliella sp.**	e.g., MK262787.1 (100%)	0	0	0.2	0.1	2.4	0	0.3	0.3	0.3	0.3	0	0.9	3.4	<	0.2	0.4	<	0	0	0	0	0	0	0	0
**Sanguina nivaloides**	e.g., MK728646.1 (100%)	0	<	0.6	1.6	0.5	10.9	0.1	0.1	0.1	0.3	0	0.3	0.8	<	<	0.3	0	<	0	0.1	0.6	0.7	0	<	<
**Staurodesmus mucronatus**	AJ428103.1 (95.4%)	0	0	0	0	0	0	0.3	<	0	0.2	0	0	0.7	0	0.1	0	0	0	0	0	0	<	0	0	0
**Stichococcus spp.**	KF144232.1 (99.1%)	0	0	0	0	0	0	0	0	0	0	0	0	0	0	0	0.8	0	0	0	0	0	0	0	0	0
**Trebouxiophyceae sp.**	e.g., MK262787.1 (99.1%)	0	0	0	0	0	0	0.2	0.4	0.4	0.2	0	0.2	1.3	<	<	0.1	0	0	0	0	0	0	0	0	0
**Trebouxiophyceae sp.**	e.g., MK262787.1 (99.7%)	0	0	0.4	<	4.1	0	0.6	0.7	0.6	0.5	<	1.5	5.8	0.2	0.3	0.9	0	0	<	0	0	0	<	0	0
**Trebouxiophyceae sp.**	e.g., MK262787.1 (99.7%)	0	0	9.7	<	12.7	<	2.4	1.1	1.0	1.8	0.2	4.1	14.3	0.5	0.6	2.1	0	0	0	0	0.2	0	0.1	<	<
**Other**		0.5	0.2	0.7	0.1	1.0	0.1	<	<	<	<	0.1	0.7	0.9	0	0.1	2.2	<	0.2	<	0.4	0.2	<	0.3	0.3	0

*****Based on light microscopic identifications in Lutz et al. (2018), this ASV likely represents *Mesotaenium* sp. (99.4% similarity with *M. berggrenii* var. *alaskana*) and not *Ancylonema nordenskioeldii* despite the slightly higher similarity (99.6%).

Supplementary Table 14. Rare Earth Element (REE) analysis concentrations ($\mu g \cdot g^{-1}$) for the mineral dust in particulate samples.

Site	Sample ID	Habitat	La	Ce	Pr	Nd	Sm	Eu	Gd	Tb	Dy	Ho	Er	Tm	Yb	Lu
3	GrIS_16_3	H_{bio} ice	34.78	66.89	7.68	28.54	4.93	1.27	4.61	0.64	3.36	0.67	1.97	0.27	1.81	0.28
	GrIS_16_10	H_{bio} ice	32.44	62.19	7.27	26.55	4.64	1.22	4.35	0.61	3.11	0.63	1.86	0.26	1.67	0.26
4a	GrIS_16_13	DCC ice	33.63	65.19	7.59	28.21	4.88	1.28	4.59	0.65	3.37	0.69	2.02	0.28	1.93	0.28
	GrIS_16_18	DCC ice	30.03	58.50	6.77	25.06	4.42	1.21	4.19	0.59	3.11	0.62	1.84	0.25	1.66	0.25
	GrIS_16_29	DCC ice	35.34	69.83	8.14	30.09	5.22	1.35	4.96	0.70	3.65	0.73	2.13	0.30	1.95	0.30
4b	GrIS_17_25	DCC ice	18.04	34.79	3.99	14.84	2.62	0.90	2.52	0.36	1.87	0.38	1.11	0.15	1.01	0.15
	GrIS_17_27	CCH	33.45	64.72	7.43	27.20	4.74	1.28	4.54	0.63	3.29	0.66	1.97	0.28	1.80	0.27
	GrIS_17_28	H_{bio} ice	17.22	32.76	3.78	14.03	2.47	0.90	2.37	0.34	1.78	0.36	1.03	0.14	0.95	0.14
	GrIS_17_31	Core CCH	31.47	61.33	7.08	26.09	4.60	1.28	4.39	0.62	3.29	0.66	1.96	0.27	1.79	0.27
	GrIS_17_34	H_{bio} ice	14.16	26.61	3.11	11.53	2.06	0.81	2.02	0.29	1.53	0.31	0.92	0.13	0.85	0.13

Supplementary Figures

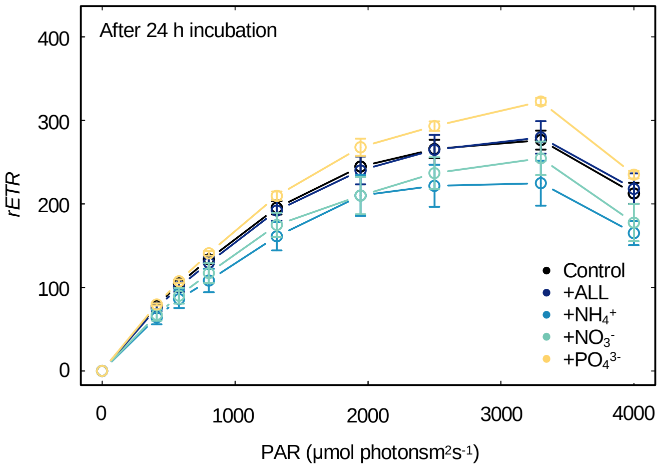

Supplementary Figure 1. Relative electron transport rates (*rETR*) measured during rapid light curves (RLCs), following a 24 h incubation. Plot shows mean ± standard error, n=4.

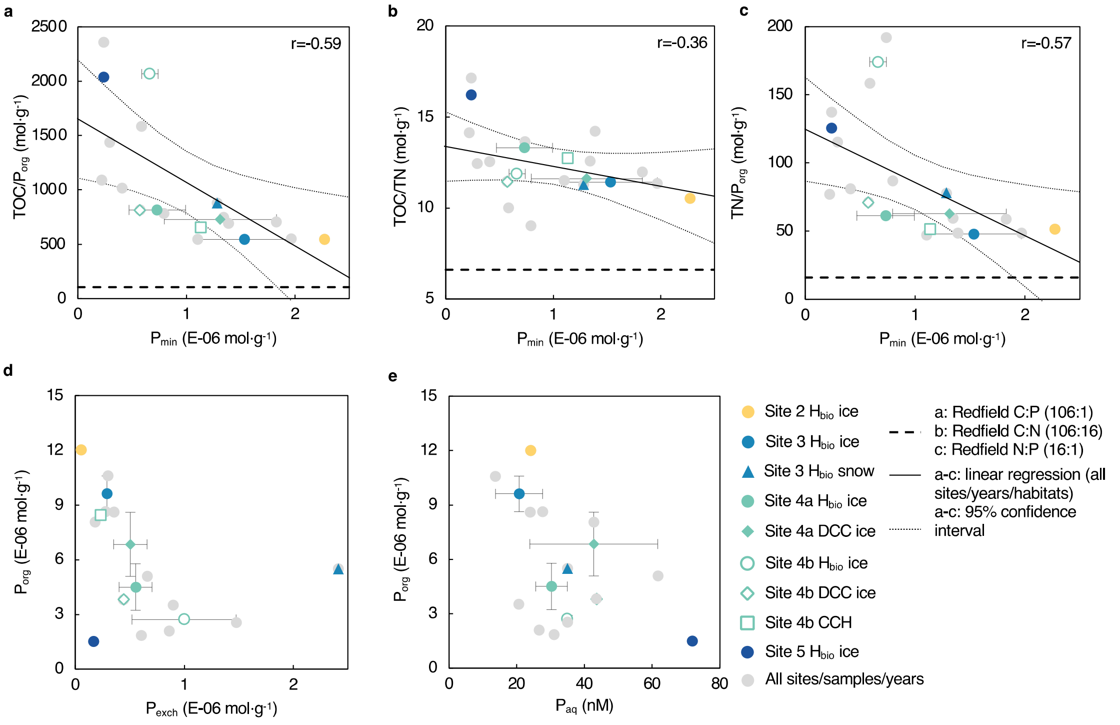

Supplementary Figure 2. P_{min} concentration in particulates plotted against a) TOC:P_{org}, b) TOC:TN, and c) TN:P_{org},. Linear regression and r-values (Pearson's product-moment correlation) correspond to all data points from all sites (grey dots). Concentrations of P_{org} in the particulate solids compared to d) solid phase P_{exch} and e) aqueous P. Colored points: mean values for different sites, habitats and years, ±SE (2016: solid fill; 2017: white-fill). In a-c: thin dashed lines: 95% confidence interval; thick dotted line indicating the respective Redfield nutrient ratio. In all plots: site 2 H_{bio} ice n= 1; Site 3 H_{bio} ice n= 2; Site 3 H_{bio} snow n= 1; Site 4a H_{bio} ice n= 5; Site 4a DCC ice n= 2; Site 4b H_{bio} ice n= 2; Site 4b DCC ice n= 1; Site 4b CCH n= 1; Site 5 H_{bio} ice n= 1.

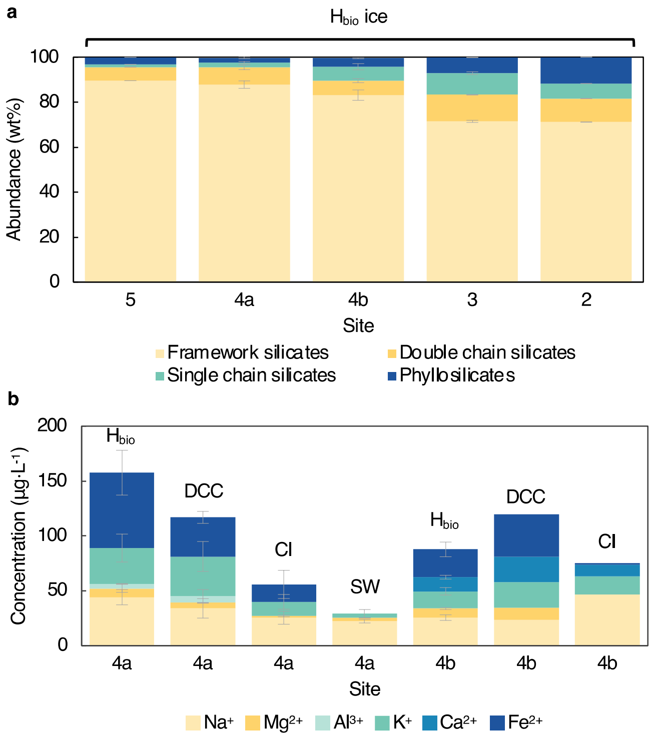

Supplementary Figure 3. a) structural mineral class abundances in high algal biomass ice (H_{bio}) ice across the ablation zone, and b) major cation concentrations in meltwater from the H_{bio} ice, dispersed cryoconite (DCC), clean ice (CI), and stream water (SW) collected in 2016 (4a) and 2017 (4b). Plot shows mean ± standard error. In a) site 5 n=1; site 4a n=5; site 4b n=4; site 3 n=2; site 2 n=1. In b) 2016: H_{bio}: n=5; DCC: n=3; CI: n=3; SW: n=2 and 2017: H_{bio}: n=3; DCC: n=1; CI: n=1.

Supplementary References

1 Williamson, C. *et al.* Algal photophysiology drives darkening and melt of the Greenland Ice Sheet. *Proc. Natl. Acad. Sci.* **117**, 5694-5705 (2020).

2 Montagnes, D. J. S., Berges, J. A., Harrison, P. J. & Taylor, F. J. R. Estimating carbon, nitrogen, protein, and chlorophyll a from volume in marine phytoplankton. *Limnol. Oceanogr.* **39**, 1044-1060 (1994).

3 Nicholes, M. J. *et al.* Bacterial Dynamics in Supraglacial Habitats of the Greenland Ice Sheet. *Front. Microbiol.* **10** (2019).

4 Bratbak, G. & Dundas, I. Bacterial dry matter content and biomass estimations. *Appl Environ Microbiol* **48**, 755-757 (1984).

5 Rudnick, R. L. & Gao, S. in *Treatise on Geochemistry, Volume 3: The Crust* Vol. 3 (eds R.L. Rudnick, H.D. Holland, & K.K. Turekian) 1-64 (Elsevier, 2003).

6 Ferrat, M. *et al.* Improved provenance tracing of Asian dust sources using rare earth elements and selected trace elements for palaeomonsoon studies on the eastern Tibetan Plateau. *Geochim. Cosmochim. Acta* **75**, 6374-6399 (2011).

7 van der Does, M., Pourmand, A., Sharifi, A. & Stuut, J.-B. W. North African mineral dust across the tropical Atlantic Ocean: Insights from dust particle size, radiogenic Sr-Nd-Hf isotopes and rare earth elements (REE). *Aeolian Res.* **33**, 106-116 (2018).

8 Tepe, N. & Bau, M. Distribution of rare earth elements and other high field strength elements in glacial meltwaters and sediments from the western Greenland Ice Sheet:

Evidence for different sources of particles and nanoparticles. *Chem. Geol.* **412**, 59-68 (2015).

9 Wientjes, I. G. M., Van de Wal, R. S. W., Reichart, G. J., Sluijs, A. & Oerlemans, J. Dust from the dark region in the western ablation zone of the Greenland ice sheet. *Cryosphere* **5**, 589-601 (2011).
