# Supplementary material for: Mineral phosphorus drives glacier algal blooms on the Greenland Ice Sheet
Source: Nat Commun. 2021 Jan 25;12:570. doi: 10.1038/s41467-020-20627-w (PMC7835244; doi:10.1038/s41467-020-20627-w)
Supplement: Supplementary file 2 — Reporting Summary [file 41467_2020_20627_MOESM2_ESM.pdf]

nature research

Corresponding author(s): Jenine McCutcheon

Last updated by author(s): Nov 26, 2020

Reporting Summary

Nature Research wishes to improve the reproducibility of the work that we publish. This form provides structure for consistency and transparency in reporting. For further information on Nature Research policies, see Authors & Referees and the Editorial Policy Checklist.

Statistics

For all statistical analyses, confirm that the following items are present in the figure legend, table legend, main text, or Methods section.

n/a	Confirmed	
☐	☒	The exact sample size (*n*) for each experimental group/condition, given as a discrete number and unit of measurement
☐	☒	A statement on whether measurements were taken from distinct samples or whether the same sample was measured repeatedly
☐	☒	The statistical test(s) used AND whether they are one- or two-sided<br>*Only common tests should be described solely by name; describe more complex techniques in the Methods section.*
☒	☐	A description of all covariates tested
☐	☒	A description of any assumptions or corrections, such as tests of normality and adjustment for multiple comparisons
☐	☒	A full description of the statistical parameters including central tendency (e.g. means) or other basic estimates (e.g. regression coefficient) AND variation (e.g. standard deviation) or associated estimates of uncertainty (e.g. confidence intervals)
☐	☒	For null hypothesis testing, the test statistic (e.g. *F*, *t*, *r*) with confidence intervals, effect sizes, degrees of freedom and *P* value noted<br>*Give P values as exact values whenever suitable.*
☒	☐	For Bayesian analysis, information on the choice of priors and Markov chain Monte Carlo settings
☒	☐	For hierarchical and complex designs, identification of the appropriate level for tests and full reporting of outcomes
☐	☒	Estimates of effect sizes (e.g. Cohen's *d*, Pearson's *r*), indicating how they were calculated

Our web collection on statistics for biologists contains articles on many of the points above.

Software and code

Policy information about availability of computer code

Data collection	No code was used to collect the data.

Data analysis

Code for processing the rapid light curves (RLC) was produced by C Williamson and is available here: https://github.com/chrisjw18/rlcs. Curve fitting was completed using R (v.3.6.0).

16S, 18S and ITS2 libraries were individually imported into Qiime2 (v.2019.1). Itsxpress was used to extract the precise ITS2 region, and thus removing the conserved regions, from the ITS2 libraries before further processing (--p-region ITS2, _--p-taxa ALL). The imported libraries were quality-filtered using the dada2 pipeline (16S: --p-trunc-len-f=280, --p-trunc-len-r=200, --p-trim-feft-f=10, --p-trim-left-r=10; 18S: --p-trunc-len-f=250, --p-trunc-len-r=200, --p-trim-feft-f=10, --p-trim-left-r=10; ITS2: --p-trunc-len-f=0, --p-trunc-len-r=0, --p-trim-feft-f=0, --p-trim-left-r=0). The amplicon sequence variants (ASV) in the filtered libraries were classified using classify-sklearn and the respective databases Greengenes (16S, "gg-13-8-99-nb-classifier"), Silva (18S, "silva-132-99-nb-classifier"), and Unite (ITS2, "unite_ver8_99_02.02.2019"). ASVs skewing the results were removed from each data set (16S: --p-exclude Chloroplast, mitochondria; 18S: --p-exclude Archaea, Bacteria). Feature tables containing solely algal (18S: --p-include Chloroplastida, Ochrophyta) or fungal (ITS2: --p-include Fungi) sequences were created. The filtered feature tables were imported into R (v.3.6.0). Non-metric multidimensional scaling (NMDS) analyses were performed using the "metaMDS" function (Bray-Curtis distances) of the R package "vegan" and plots were created using the package "ggplot2". Analysis of similarities (ANOSIM) was carried out using the "anosim" function of the "vegan" package and "sites" and "habitats" as treatment groups.

The ggplot2 package (v3.2.1 https://cran.r-project.org/web/packages/ggplot2/readme/README.html) was used in R (v3.6.0) to process and plot the particle size distribution data.

X-ray diffraction data was processed using Bruker DIFFRACplus Eva (v.2) and Topas (v4.2) software.

Pearson's product-moment correlation r-values were calculated using Excel (v16.xx).

For manuscripts utilizing custom algorithms or software that are central to the research but not yet described in published literature, software must be made available to editors/reviewers. We strongly encourage code deposition in a community repository (e.g. GitHub). See the Nature Research guidelines for submitting code & software for further information.

Data

Policy information about availability of data

All manuscripts must include a data availability statement. This statement should provide the following information, where applicable:

- Accession codes, unique identifiers, or web links for publicly available datasets
- A list of figures that have associated raw data
- A description of any restrictions on data availability

Detailed microbial community, and fluid and solid phase chemistry results are available in the supplementary information file. The microbial community data is available through the sequence read archive under accession number PRJNA564214. The COD database is available here: http://www.crystallography.net/cod/. Figures that have associated raw data: 2,3,4,6.

Field-specific reporting

Please select the one below that is the best fit for your research. If you are not sure, read the appropriate sections before making your selection.

☐ Life sciences ☐ Behavioural & social sciences ☒ Ecological, evolutionary & environmental sciences

For a reference copy of the document with all sections, see nature.com/documents/nr-reporting-summary-flat.pdf

Ecological, evolutionary & environmental sciences study design

All studies must disclose on these points even when the disclosure is negative.

Study description

This study characterizes the mineralogy, geochemistry, and microbial community composition of glacier algal habitats in the Dark Zone of the southwest Greenland Ice Sheet. A nutrient addition experiment was used to demonstrate that phosphorus is a limiting nutrient for glacier algae in the Dark Zone and identify phosphorus-bearing minerals as a potential nutrient source fueling glacier algal blooms. Details of the study design structure and numbers of replicates are listed in the methods section of the manuscript.
Briefly:
Nutrient incubation experiment: n=5 Hbio ice samples collected at site 4a; the 5 treatments run as n=4.
Sequential P extraction: site 2 Hbio ice n= 1; site 3 Hbio ice n= 2; site 3 Hbio snow n= 1; site 4a Hbio ice n= 5; site 4a DCC ice n= 2; site 4b Hbio ice n= 2; Site 4b DCC ice n= 1; Site 4b CCH n= 1; Site 5 Hbio ice n= 1.
C and N analysis: site 2 Hbio ice n=1; site 3 Hbio ice n=2, Hbio snow n=1; Site 4a Hbio ice n=6, DCC ice n= 4; site 4b Hbio ice n=2, DCC ice n=1; cryoconite hole n=1; site 5 Hbio ice n=2.
X-ray diffraction: site 2 Hbio ice n=1; site 3 Hbio ice n=3, site 4a Hbio ice n=5, DCC ice n=4; site 4b Hbio ice n=4, DCC ice n=1, cryoconite n=1; site 5 Hbio n=1.
Fluid chemistry: site 1 clean snow n=1; site 2 Hbio ice n=1; site 3 Hbio ice n=2, Hbio snow n=2; site 4a Hbio ice n=5, DCC ice n=3, clean ice n=3, supraglacial stream water n=2; site 4b Hbio ice n=3, DCC ice n=1, clean ice n=1; site 5 Hbio ice n=5.
Algal, fungal, and bacterial community composition: n=26.
Scanning electron microscopy: n=12 Hbio ice samples.
Particle size analysis: n=24 Hbio.
Particle mass loading: clean ice: n= 4 samples, Hbio ice: n= 3 samples, DCC ice: n=1 sample.
Rare Earth element analysis: rocks n=4, ice n=10.

Research sample

Samples of glacier algae and associated heterotrophic bacteria and fungi were collected from the ice surface. Melted snow and ice,

Research sample	and supraglacial meltwater were preserved to characterize the fluid chemistry of the glacier algal habitat. Solid materials, namely mineral dust, were collected from melted samples. Samples were selected to represent the range of ice surface habitats present on the ice sheet during the summer melt season.
Sampling strategy	Surface snow and ice samples were collected along a transect across the ablation zone of the southwestern margin of the Greenland Ice Sheet during the 2016 (July 27 – August 17) and 2017 (June 1 – 28) melt seasons. Ice and snow samples (1 – 10 L) were collected into sterile plastic bags, melted at ambient temperatures (5-10 degrees C). No sample-size calculation was performed. Sample sizes were dependent on site access, field conditions, and time frames required for sampling and processing samples in the field.<br>Reference rocks were collected near the Russell Glacier terminus in 2018. Sample sizes were selected to be as large as possible given the time restrictions of site access and sample processing on the ice sheet. Where possible, samples were collected as n=3 or more. A sample size of n=3 is typically considered acceptable during remote field campaigns. We have included data for some additional sample types that are of interest but for which n<3. Such instances are noted in the study (e.g. the biofilm).
Data collection	pH was measured in the field by J. McCutcheon, S. Lutz, L.G .Benning, and J.B. McQuaid using an Orion™ Star A321 pH meter. Conductivity and total dissolved solids were measured using an Orion Star™ A222 meter with a corresponding <100 µS/cm2 conductivity probe. pH, conductivity, and total dissolved soloids data was recorded by J. McCutcheon and S. Lutz.<br>Rapid light curve data was collected by C. Williamson and A. Anesio using a WaterPAM fluorometer and attached red-light emitter/ detector cuvette system (Walz GmbH, Germany).<br>Scanning electron microscopy data was collected by J. McCutcheon using a Hitatchi 8230 SEM at the Leeds Electron Microscopy and Spectroscopy Centre (LEMAS), University of Leeds, UK.<br>X-ray diffraction was conducted by J. McCutcheon using a Bruker D8 Advance Eco X-ray diffractometer (Bruker, Billerica, USA) with a Cu source at the University of Leeds, UK.<br>ICP-MS was conducted by S. Reid using a Thermo Fisher iCAPQc ICP-MS at the University of Leeds, UK.<br>Phosphorus was measured either using segmented flow-injection analysis (AutoAnalyser3, Seal Analytical), or for samples containing lower concentrations of phosphorus by A. Stockdale, using a 100 cm WPI Liquid Waveguide Capillary Cell in conjunction with an Ocean Optics USB2000+ spectrophotometer. Both analyses were conducted at the University of Leeds, UK.<br>Ion chromatography was conducted by A. Viet-Hillebrand at the German Research Centre for Geosciences, Potsdam, Germany using a conductivity detector on a Dionex ICS 3000 system, equipped with an AS 11 HC Dionex analytical column.<br>Carbon and nitrogen analysis was conducted by B. Plessen and S. Pinkerneil at German Research Centre for Geosciences, Potsdam, Germany using an NC2500 Carlo Erba elemental analyzer.<br>Amplicon libraries were sequenced on the Illumina MiSeq using paired 300-bp reads at the University of Bristol Genomics Facility, Bristol, UK.<br>Rare Earth element concentrations were measured by A. Vanderstraeten using HR-ICP-MS (ThermoFisher Element 2) at the Vrije Universiteit Brussel, Belgium.<br>Particle size distribution was measured by K. Jurkschat using a DC24000 CPS disc centrifuge at Oxford Materials Characterisation Services, Oxford, UK.
Timing and spatial scale	Timing: data were collected in the field from July 27 – August 17, 2016 and June 1 – 28. 2017. These dates were selected to have data from consecutive arctic summers. When possible, samples of high algal biomass ice, dispersed cryoconite ice, and clean ice were collected in sets on a single day in close proximity to each other (< 10 m apart). This was conducted every 3–5 days, with the interceding days used to melt and process each set of samples. Nutrient addition experiment sampling frequency was selected based on the anticipated time frame required to observe a photophysiological response by the glacier algae. Laboratory measurements (ICP-MS, IC, XRD, REE, CPS, SEM, carbon and nitrogen analysis) were conducted between September 2016 and July 2019.<br><br>Spatial scale: Samples were collected along a 97 km long transect. Sample locations were located at 150, 75, 35, and 33 km from the ice sheet margin. At each location, all samples were collected within an area of 1 km2.
Data exclusions	No data were excluded from the study.
Reproducibility	Data from sites 1,2,3,5, and 6 were not reproducible, due to these sites only being visited once. Site 4 was sampled multiple times both in 2016 and 2017 to confirm reproducible trends at this location. On a melting ice sheet reproducible sampling from year to year is not possible.
Randomization	Solid phase samples were homogenized and an aliquot of material from each sample was randomly selected for each analysis. Ice and snow samples were fully melted and an aliquot of sample was randomly selected for analysis.
Blinding	Blinding was not used during sample collection. Blinding is not a common practice in field studies, and is generally not possible. Surface ice habitat observations are necessary for accurate and consistent sample collection. During analyses in the laboratory, samples were labeled with only a number and thus investigators did not know which sample they were analyzing.

Did the study involve field work? ☒ Yes ☐ No

Field work, collection and transport

Field conditions	Fieldwork was conducted on the Greenland Ice Sheet. Ground conditions consisted of stable bare ice, occasionally covered over with snow. Weather conditions consisted of air temperatures between -8 and 8 degrees C, intermittent snow and rainfall, and katabatic winds from the southeast. Both expeditions were conducted during periods of 24 h daylight.
Location	Location: 67.04N, 49.07W<br>Elevation: 1100 m
Access and import/export	Field locations were accessed via helicopter from Kangerlussuaq. Expedition permits were obtained from the Government of Greenland for the 2016 (KNNO Expedition Permit C-16-25, 29/06/2016) and 2017 (KNNO Expedition Permit C-17-24,

	23/05/2017) field seasons. Biological and soil samples were collected and imported on permits issued by the Landesamt für Ländliche Entwicklung, Landwirtschaft und Flurneuordnung Pflanzengesundheitskontrolle (permit: 2008/61/EG, 5/10/2017).
Disturbance	All fieldwork was conducted from a temporary basecamp consisting of 7-12 tents. The camp was primarily powered by solar panels, with a generator used only intermittently. All equipment and rubbish were removed from the site at the conclusion of each expedition. No disturbance was caused by the study.

Reporting for specific materials, systems and methods

We require information from authors about some types of materials, experimental systems and methods used in many studies. Here, indicate whether each material, system or method listed is relevant to your study. If you are not sure if a list item applies to your research, read the appropriate section before selecting a response.

Materials & experimental systems

n/a	Involved in the study
☒	☐ Antibodies
☒	☐ Eukaryotic cell lines
☒	☐ Palaeontology
☒	☐ Animals and other organisms
☒	☐ Human research participants
☒	☐ Clinical data

Methods

n/a	Involved in the study
☒	☐ ChIP-seq
☒	☐ Flow cytometry
☒	☐ MRI-based neuroimaging
